# The diagnostic accuracy of intraoperative frozen section biopsy for diagnosis of sentinel lymph node metastasis in breast cancer patients: a meta-analysis

**DOI:** 10.1007/s11356-022-20569-4

**Published:** 2022-05-11

**Authors:** Alaa Ahmed Elshanbary, Alaa Abdelsameia Awad, Alaa Abdelsalam, Islam H. Ibrahim, Walid Abdel-Aziz, Youssef Bahaaeldin Darwish, Alaa Saad Isa, Boutheyna Drid, Marwa Gamal Mustafa, Radwa Hamdy Allam, Amira A. Abo Ali, Anas Zakarya Nourelden, Khaled Mohamed Ragab, Hussah I. M. AlGwaiz, Aeshah A. Awaji, Mousa O. Germoush, Ashraf Albrakati, Marina Piscopo, Nehmat Ghaboura, Mohamed Sayed Zaazouee

**Affiliations:** 1 Faculty of Medicine, Alexandria University, Alexandria, Egypt; 2 International Medical Research Association (IMedRA), Cairo, Egypt; 3 Faculty of Medicine, Zagazig University, Zagazig, Egypt; 4 Faculty of Medicine, Assiut University, Assiut, Egypt; 5 Faculty of Medicine, Al-Azhar University, Assiut, Egypt; 6 Faculty of Pharmacy, Mansoura University, Mansoura, Egypt; 7 Faculty of Medicine, Al-Azhar University, Damietta, Egypt; 8 Faculty of Medicine, Batna 2 University, Batna, Algeria; 9 Faculty of Pharmacy, Fayoum University, Fayoum, Egypt; 10 Faculty of Medicine, Fayoum University, Fayoum, Egypt; 11 Faculty of Medicine, Al-Azhar University, Cairo, Egypt; 12 Faculty of Medicine, Minia University, Minia, Egypt; 13 Department of Biology, College of Science, Princess Nourah Bint Abdulrahman University, Riyadh, 11474 Saudi Arabia; 14 Department of Biology, Faculty of Science, University College of Taymaa, University of Tabuk, Tabuk, 71491 Saudi Arabia; 15 Biology Department, College of Science, Jouf University, P.O. Box: 2014, Sakaka, Saudi Arabia; 16 Department of Human Anatomy, College of Medicine, Taif University, P.O. Box 11099, Taif, 21944 Saudi Arabia; 17 Department of Biology, University of Naples Federico II, Via Cinthia, 80126 Naples, Italy; 18 Department of Pharmacy Practice, Batterjee Medical College, Pharmacy Program, P.O. Box 6231, Jeddah, 21442 Saudi Arabia

**Keywords:** Breast cancer, Sentinel lymph node, Frozen section biopsy, Meta-analysis, Intraoperative evaluation

## Abstract

Sentinel lymph node (SLN) sampling is important for evaluating the nodal stage of breast cancer when the axillary nodes are clinically free of metastasis. The intraoperative frozen section (IFS) of SLN is used for lymph node assessment. This meta-analysis aims to provide evidence about the diagnostic accuracy and the applicability of IFS of SLN in breast cancer patients. Data were collected by searching PubMed, Cochrane, Scopus, and Web of Science electronic databases for trials matching our eligibility criteria. The statistical analysis included the sensitivity, specificity, positive likelihood ratio, negative likelihood ratio, and pooled studies’ diagnostic odds ratio outcomes. The analyses were conducted using the Open Meta-analyst software. This meta-analysis pooled the results of 110 studies. The overall sensitivity of IFS for SLN metastasis was 74.7%; 95% CI [72.0, 77.2], *P* < 0.001. It was 31.4% 95% CI [25.2, 38.3], *P* < 0.001 for the micro-metastasis, and 90.2%; 95% CI [86.5, 93.0], *P* < 0.001 for the macro-metastasis. The overall specificity was 99.4%; 95% CI [99.2, 99.6], *P* < 0.001. The overall positive likelihood ratio was 121.4; 95% CI [87.9, 167.6], *P* < 0.001, and the overall negative likelihood ratio was 0.226; 95% CI [0.186, 0.274], *P* < 0.001. The overall diagnostic odds ratio of IFS for diagnosing SLN metastasis was 569.5; 95% CI [404.2, 802.4], *P* < 0.001. The intraoperative frozen section of SLN has good sensitivity for diagnosing breast cancer macro-metastasis. However, the sensitivity is low for micro-metastasis. The specificity is very satisfactory.

**Supplementary Information:**

The online version contains supplementary material available at 10.1007/s11356-022-20569-4.

## Introduction

Recently cancer statistics stated that breast cancer has the highest incidence among all cancers in women. In 2020, more than 680,000 died from breast cancer worldwide (Sung et al. 2021). Due to recently developed screening programs and elevated public awareness of breast cancer, the early detection rate has increased; therefore, axillary metastases’ incidence rates are continuously decreasing (Berry et al. 2005).

The sentinel lymph node biopsy (SLNB) technique is routinely used for nodal staging of breast cancer when nodal metastasis is not manifested clinically. Lymph node evaluation is essential because axillary lymph nodes’ status affects survival and the cancer recurrence rate more than any other factor (Fisher et al. 1983). SLNB has a lower incidence of complications, especially lymphedema, than axillary lymph node dissection (ALND). This limits the use of ALND only to patients with metastatic sentinel lymph nodes (SLN) (Cipolla et al., 2010; Lucci et al. 2007; Peintinger et al. 2003; Veronesi et al. 2003).

Intraoperative frozen section (IFS) is one of the most commonly used methods for intraoperative assessment of SLN. If intraoperative SLN is positive, patients will proceed directly for immediate ALND, thus sparing them from the burden of a second operation which may be more complex, time-consuming, and carry greater risks from anesthesia or other possible complications (Veronesi et al. 2003).

The American College of Surgeons Oncology Group Z0011 trial (ACOSOGZ0011) started in the late 1990s and the International Breast Cancer Study Group 23–01 trial (IBCSG 23–01) started in 2001 revised the indications to perform ALND in positive SLN patients. As many positive SLN cancer patients do not have additional metastatic lymph nodes at the ALND, the ACOSOG Z0011 trial randomized patients with T1 to T2 tumors and (1–2) positive SLNs who underwent conservative breast surgery with whole-breast irradiation to either complete the ALND or to not undergo any further axillary surgery. ACOSOGZ0011 showed no differences in overall 10-year survival between the patients treated with ALND and those treated with SLNB alone with less morbidity in SLNB. Also, results from IBCSG 23–01 after a 10-year follow-up showed that ALND could be safely carried out in T1 to T2 breast cancer patients with SLN micro-metastases (Baron et al. 2007; Galimberti et al. 2013; Giuliano 2011; Giuliano et al. 2017). According to the American Society of Clinical Oncology, IFS is the recommended method for the intraoperative evaluation of SLNs. However, IFS may cause some destruction to the diagnostic tissue (Lyman et al. 2005).

Several studies investigated the applicability of IFS in detecting macro-metastasis (MAM) and micro-metastasis (Mi) of SLNs, but with significant variability in their samples and results. Furthermore, many studies have been published since the last meta-analysis determined the IFS applicability, so we aim to provide a current, complete vision about the overall accuracy and applicability of IFS of SLNs in breast cancer patients.

## Methods

We adopted the Preferred Reporting Items for Systematic Reviews and Meta-Analyses (PRISMA) statement (Liberati et al., 2009). We followed the guidelines of the “Cochrane Handbook for Systematic Reviews of Diagnostic Test Accuracy” (Macaskill et al., 2010).

### Literature search

We searched the published literature on PubMed, Cochrane, Scopus, and Web of Science databases using the following keywords: “sentinel lymph node,” “SLN,” “frozen section biopsy,” “breast cancer,” “breast neoplasm,” “mammary cancer,” “breast tumor,” and “breast carcinoma.” The last search update was in January 2021. After removing duplicates by Endnote, four authors screened titles and abstracts of retrieved records according to our eligibility criteria. Then, potentially eligible articles underwent full-text screening to confirm their eligibility for the meta-analysis. In addition, we searched references of included studies manually for additional relevant articles according to our eligibility criteria. Any discrepancy among authors was solved by discussion and consensus. Two reviewers revised the screening process to ensure that all eligible studies were included.

### Study eligibility criteria

We included observational and interventional trials on breast cancer that enrolled patients with no clinical manifestations of nodal metastasis. Studies that compared the diagnostic accuracy of IFS for SLN metastasis with that of definitive histopathology were included. We applied no restrictions concerning language, publication date, place, or age. For overlapping datasets, we included the articles reporting the most complete data set. We excluded articles with missing sensitivity or specificity data. We also excluded reviews, letters, editorials, conference papers, and animal studies.

### Data extraction

Five authors extracted the following data items in a separate Excel sheet: (1) summary of the included studies, including design, sample size, number of SLNB, reference comparator, and conclusions; (2) baseline criteria of included population, including age, mean number of SLN, radiology tumor size, histologic type, estrogen and progesterone receptors, human epidermal growth factor receptor 2, lympho-vascular invasion, nuclear grade, and type of metastasis; and (3) diagnostic accuracy outcomes, including true positive, true negative, false positive, and false negative. When different authors found differences in extracted data, these disagreements were solved by discussion and consensus.

### Quality assessment of included studies

To assess the quality of the included studies, we used the quality assessment tool for the diagnostic accuracy studies (QUADAS) (Whiting et al. 2003). This tool includes the risk of bias and applicability concerns of the following items: (1) patient selection, including three risk of bias domains: random or consecutive sampling, case–control design avoidance, and inappropriate exclusions avoidance; (2) index test, including two risk of bias domains: blinding of the reference standard results during index test result interpretation, and pre-specification of the used threshold if present; and (3) reference standard, including two risk of bias domains: the correct classification of the target condition by the reference standard, and blinding of the index test results during reference standard result interpretation. In addition, the tool includes the risk of bias for another item: (4) flow and timing, including three domains: the appropriate interval between index test and reference standard, including all patients in the analysis, and receiving the same reference standard by all patients. Each risk of bias item was judged as low, high, or unclear risk of bias, and each applicability concern item was judged as low, high, or unclear concern. Five authors assessed the risk of bias independently, and any disagreement was resolved by discussion and a senior reviewer consultation.

### Statistical analysis

We used the Open Meta-analyst software to execute our analyses. To assess the diagnostic accuracy of IFS, we calculated its sensitivity, specificity, positive likelihood ratio (PLR), negative likelihood ratio (NLR), and diagnostic odds ratio (DOR) with 95% confidence intervals (CIs). Besides, we used the summary receiver operating characteristic (SROC) curve analyses with the sensitivity representing the *Y*-axis and the 1-specificity representing the *X*-axis. We pooled the results of included studies using the DerSimonian-Laird method under the random-effects model. We assessed heterogeneity across studies using the chi-square test and evaluated its extent using the *I*-square test. Heterogeneity was considered significant when the chi-square *P*-value was less than 0.1 and *I*^2^ > 50%. We conducted the analyses for these outcomes on the total sample. In addition, we analyzed the sensitivity and the DOR outcomes for MAM and Mi separately. Isolated tumor cell implants were considered as Mi metastases.

## Results

### Literature search results

**Fig. 1:**
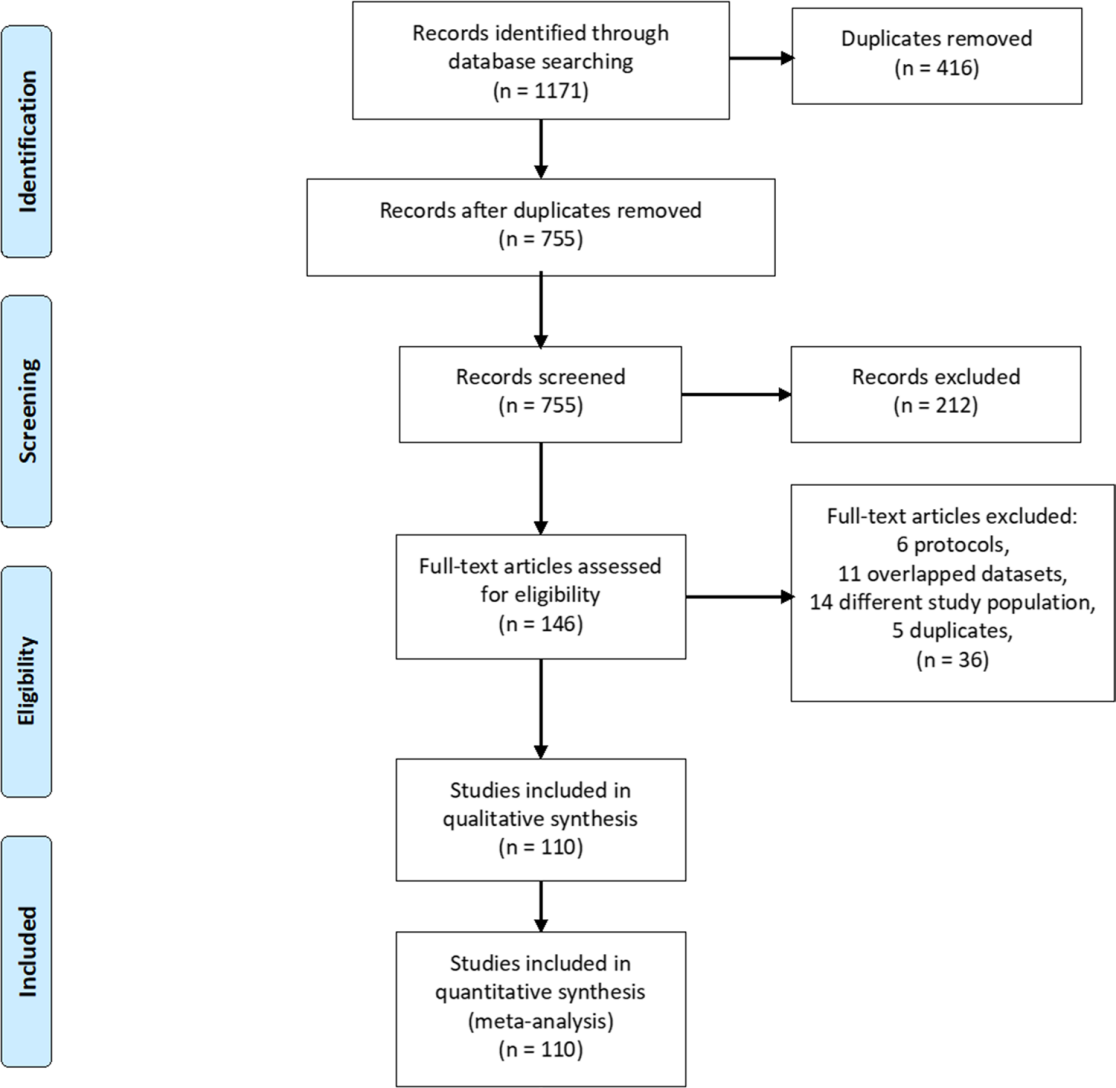
The PRISMA flow diagram showing the steps of data collection, screening, and selection of the included studies

We retrieved 755 unique records from searching databases. After title and abstract screening, 146 studies were subjected to full-text screening. Among these studies, only 110 studies were eligible for the analysis. The PRISMA flow diagram shows the details of the data collection, screening, and study selection process (Fig. 1).

### Characteristics of included studies and study population

We included 110 studies (62 prospective and 48 retrospective) with a total of 47,622 patients and about 65,532 SLNB. The mean age of subjects in included studies varied from 45 ± 9 to 65 ± 8 years. Infiltrating ductal carcinoma was the commonest histological type of breast cancer, and most patients had positive estrogen and positive progesterone receptors. Supplementary Table 1 shows the summary of included studies, and Supplementary Table 2 shows the features of their included subjects.

### Quality assessment

**Fig. 2:**
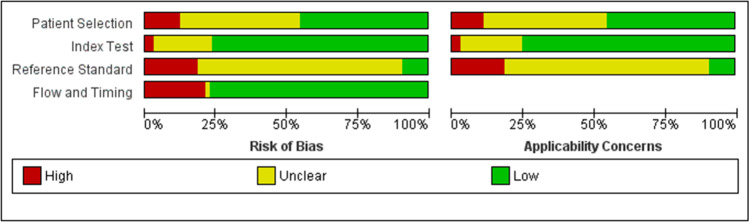
The risk of bias graph, precisely showing each quality assessment item’s overall judgment

Regarding the risk of bias, most studies had a low risk of bias concerning index test and flow and timing items. About half of the studies had a low risk of bias as for patient selection. Most studies had an unclear risk of bias about reference standard. Regarding applicability concerns, most studies had a low risk of bias as for index test and an unclear risk of bias as for reference standard. About half of the studies had a low risk of bias regarding patient selection. Figure 2 shows the recap of the quality assessment items, and Supplementary Fig. 1 shows the detailed judgment of each item.

### Study outcomes

#### ***Sensitivity***

**Fig. 3:**
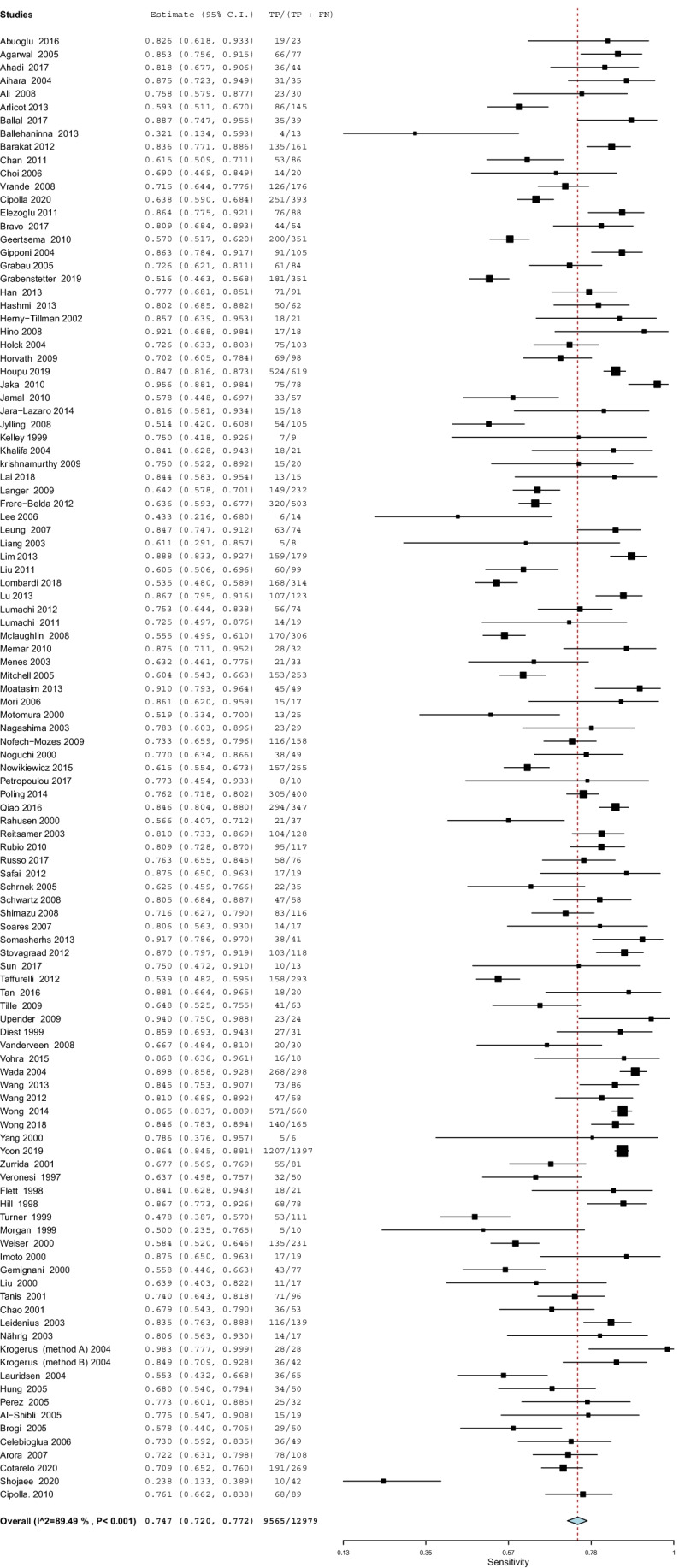
A forest plot for the pooled sensitivity of intraoperative frozen section biopsy to detect sentinel lymph node metastasis in breast cancer patients

**Fig. 4:**
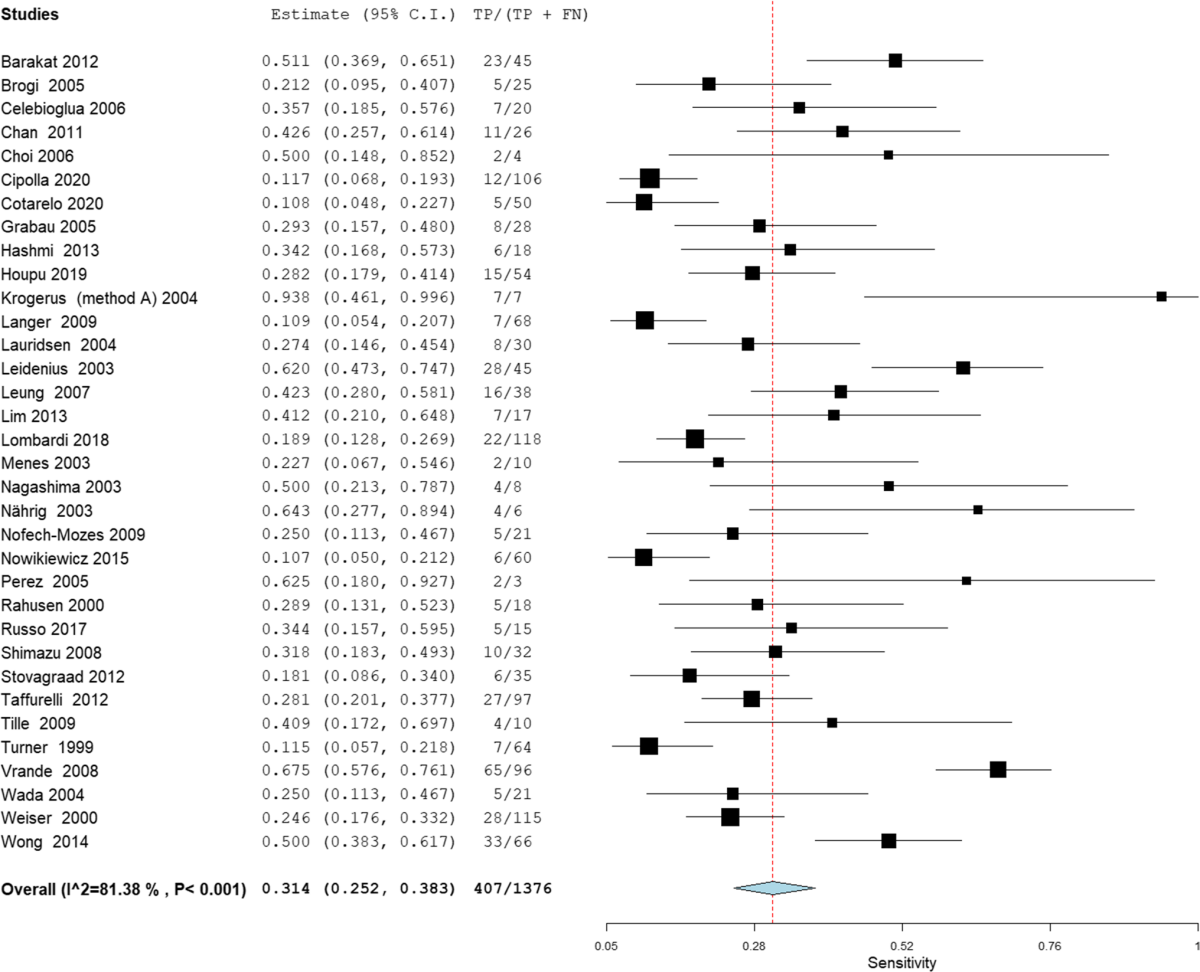
A forest plot for the pooled sensitivity of intraoperative frozen section biopsy to detect sentinel lymph node micro-metastasis in breast cancer patients

**Fig. 5:**
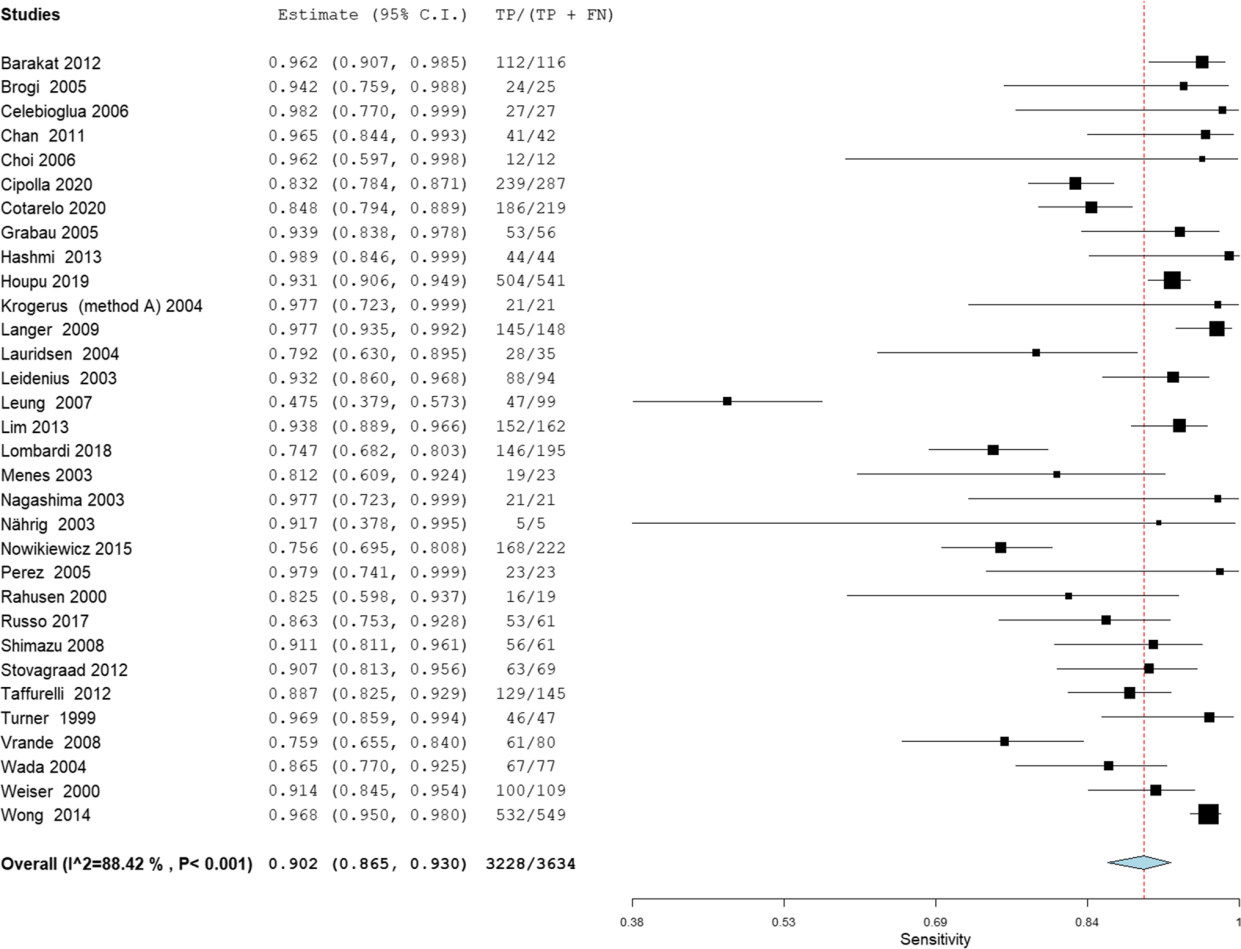
A forest plot for the pooled sensitivity of intraoperative frozen section biopsy to detect sentinel lymph node macro-metastasis in breast cancer patients

The overall sensitivity of IFS for detection of SLN metastasis was 74.7%; 95% CI [72.0, 77.2], *P* < 0.001 (Fig. 3). Pooled studies were heterogenous (*P* < 0.001; *I*^2^ = 89.5%). The overall sensitivity of IFS for detection of SLN Mi was 31.4%; 95% CI [25.2, 38.3], *P* < 0.001 (Fig. 4). Pooled studies were heterogenous (*P* < 0.001; *I*^2^ = 81.4%). The overall sensitivity of IFS for detection of SLN MAM was 90.2%; 95% CI [86.5, 93.0], *P* < 0.001 (Fig. 5). Pooled studies were heterogenous (*P* < 0.001; *I*^2^ = 88.4%).

#### Specificity

**Fig. 6:**
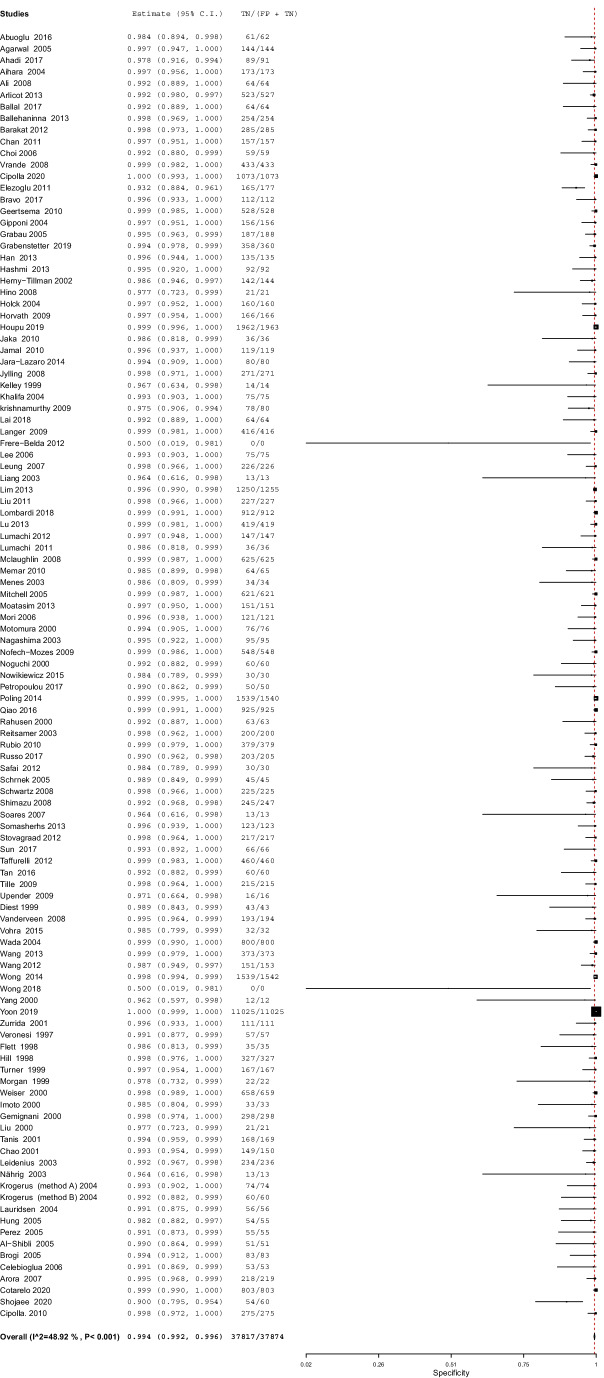
A forest plot for the pooled specificity of intraoperative frozen section biopsy in diagnosing sentinel lymph node metastasis in breast cancer patients

**Fig. 7:**
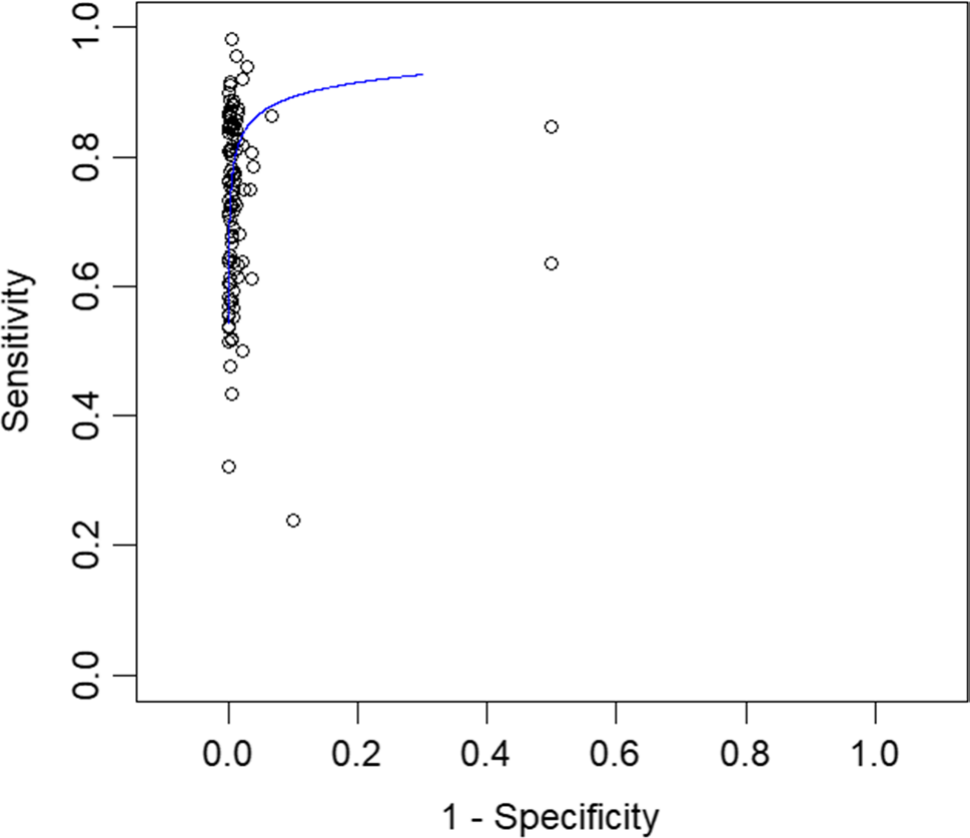
The summary receiver operating characteristic (SROC) curve

The overall specificity of IFS for detection of SLN metastasis was 99.4%; 95% CI [99.2, 99.6], *P* < 0.001 (Fig. 6). Pooled studies were heterogenous (*p* < 0.001; *I*^2^ = 48.9%). The SROC curve shows the trade-off between sensitivity and 1-specificcity (Fig. 7).

#### Positive likelihood ratio

The overall positive likelihood ratio of IFS for detection of SLN metastasis was 121.4; 95% CI [87.9, 167.6], *P* < 0.001 (Supplementary Fig. 2). Pooled studies were heterogenous (*P* < 0.001; *I*^2^ = 56.5%).

#### Negative likelihood ratio

The overall negative likelihood ratio of IFS for detection of SLN metastasis was 0.226; 95% CI [0.186, 0.274], *P* < 0.001 (Supplementary Fig. 2). Pooled studies were homogenous (*P* = 1; *I*^2^ = 0%).

#### Diagnostic odds ratio

The overall diagnostic odds ratio for diagnosis of SLN metastasis by IFS was 569.5; 95% CI [404.2, 802.4], *P* < 0.001 (Supplementary Fig. 3). Pooled studies were heterogenous (*P* < 0.001, *I*^2^ = 54%). The overall diagnostic odds ratio for diagnosis of SLN Mi by IFS was 1.347; 95% CI [0.468, 3.882], *P* = 0.581 (Supplementary Fig. 4). Pooled studies were heterogenous (*P* < 0.001, *I*^2^ = 62.6%). The overall odds ratio for diagnosis of SLN MAM by IFS was 29.245; 95% CI [10.29, 83.114], *P* < 0.001 (Supplementary Fig. 5). Pooled studies were heterogenous (*P* < 0.001, *I*^2^ = 60.1%).

## Discussion

Our analysis showed that the IFS of SLN, which is used to diagnose breast cancer lymph node metastasis, has a sensitivity of 74.7% and a specificity of 99.4%. Patients with positive test results have 121-fold higher odds of having SLN metastasis (positive likelihood ratio), while patients with negative test results have 4.4-fold lower odds of having SLN metastasis (negative likelihood ratio). By subgroup analysis, we also found that sensitivity was 31.4% in terms of micro-metastasis, while regarding macro-metastasis, sensitivity was 90.2%.

When metastasis proceeds, it first affects the axillary lymph nodes (ALNs). Thus, breast cancer may be first suspected by detecting the clinically affected nodes. Therefore, ALN metastasis is considered an indicator of overall recurrence and survival rates (Huston and Simmons 2005). Precise assessment of ALN yields information about the stage of breast cancer or provides instructions concerning treatment options. Surgical management has progressed from radical resection to further advanced procedures and strategies (Coughlin and Ekwueme 2009; Samphao et al. 2008). ALND is a prognostic and therapeutic index that is one of the initial approaches for managing clinically positive nodes in breast cancer patients. However, ALND may result in numerous side effects such as shoulder mobility disorders, wound infections, and seroma formation (Roses et al. 1999). Accordingly, SLNB has displaced ALND in detecting lymph node metastases in order to avoid ALND side effects and complications (Schrenk et al., 2000).

SLNB provides a more accurate diagnostic method with a low false-negative rate and corresponding lower morbidity rates (McMasters et al. 2000). If intraoperative SLN analysis is free from metastasis, ALND can then be avoided, but if the result is positive, ALND is performed during the tumor removal, thus avoiding the need for a second surgery. Moreover, the pathologist can obtain diverse details by examining only a small number of nodes concerning SLN (Cserni et al., 2004, 2003).

Various techniques were mentioned in the literature for intraoperative assessment of SLN, such as IFS analysis, touch imprint cytology (TIC), and rapid cytokeratin immunostaining or combinations of these procedures (Madsen et al. 2012).

An IFS is most often used in oncological surgery such as breast cancer, lung, and endometrial surgeries. Luis Alcazar et al. reported that in patients with endometrial cancer, IFS was superior to intraoperative gross evaluation (IGE) for diagnosing deep myometrial malignant infiltration (Alcazar et al. 2016). Also, IFS may evaluate the extent of local malignant infiltration in patients with lung adenocarcinoma. It has high accuracy and ability to differentiate between pre-/minimally invasive adenocarcinoma (IAC) and IAC (Li et al. 2019).

However, IFS has several disadvantages, including loss of tissue during the sectioning process, tissue architecture alteration, tissue manipulation due to freezing and resoftening of specimens, and the high cost (Martínez García, 2019; Treseler 2006; Varga et al. 2008).

However, using IFS of SLNB in breast cancer is still questionable due to the notable variation in its sensitivity. Previous studies revealed that IFS sensitivity in identifying Mi is low compared to MAM (Morgan et al. 1999; Veronesi et al. 1996; Weiser et al. 2000). In 2012, a previous meta-analysis reported that, by pooling the results of 47 studies comprising 13,062 women with breast cancer, the IFS of SLNs has an outstanding sensitivity for MAM, reaching 94%. However, it was not sensitive enough for Mi at a level of 40%. The mean specificity was 100% (Liu et al. 2011). Our results are similar to their results, with a sensitivity of 31.4% for MAM, 90.2% for Mi, and a specificity level reaching 99.4% that included 47 studies comprising 13,062 women.

Our meta-analysis followed the steps described in the “Cochrane Handbook for Systematic Reviews of Diagnostic Test Accuracy.” We included many studies with an overall large sample size which increased the generalizability of our results. All study designs were included. We pooled all studies together then subgrouped them into either Mi or MAM to detect the sensitivity for both types. In general, the included studies had a moderate quality. However, our study has some limitations as most of the included studies were observational. Also, significant heterogeneity was detected.

Reliable intraoperative techniques for detecting SLN micro-metastasis are still lacking. We recommend future studies to conduct a network meta-analysis to compare the diagnostic accuracy of different diagnostic techniques.

We concluded that, for the diagnosis of metastasis caused by breast cancer, the sensitivity of IFS has excellent sensitivity (90.2%) for macro-metastasis detection in SLNs, while the sensitivity for the diagnosis of micro-metastasis is lower (31.4%). The overall specificity is satisfying (99.4%).

## Supplementary Information

Below is the link to the electronic supplementary material.

Supplementary Fig. 1: The risk of bias summary, showing each quality assessment item’s judgment in each study. (PDF 135 KB)

Supplementary Fig. 2: Forest plots for the pooled negative likelihood ratio (to the left) and positive likelihood ratio (to the right) of intraoperative frozen section biopsy in detecting sentinel lymph node metastasis in breast cancer patients. (PDF 19 KB)

Supplementary Fig. 3: A forest blot for the pooled diagnostic odds ratio of intraoperative frozen section biopsy in the detection of sentinel lymph node metastasis in breast cancer patients. (PDF 12 KB)

Supplementary Fig. 4: A forest blot for the pooled diagnostic odds ratio of intraoperative frozen section biopsy in the detection of sentinel lymph node micro-metastasis in breast cancer patients. (PDF 7 KB)

Supplementary Fig. 5: A forest blot for the pooled diagnostic odds ratio of intraoperative frozen section biopsy in detecting sentinel lymph node macro-metastasis in breast cancer patients. (PDF 7 KB)

Supplementary file6 (DOCX 263 KB)

## Data Availability

The data that support the findings of this study are available from the corresponding author upon reasonable request.
