# Supplementary material for: The diagnostic accuracy of intraoperative frozen section biopsy for diagnosis of sentinel lymph node metastasis in breast cancer patients: a meta-analysis
Source: Environ Sci Pollut Res Int. 2022 May 11;29(32):47931–41. doi: 10.1007/s11356-022-20569-4 (PMC9252966; doi:10.1007/s11356-022-20569-4)
Supplement: Supplementary file 1 — Supplementary Fig. 1: The risk of bias summary, showing each quality assessment item’s judgment in each study. (PDF 135 KB) [file 11356_2022_20569_MOESM1_ESM.pdf]

Study	Risk of Bias				Applicability Concerns		
	Patient Selection	Index Test	Reference Standard	Flow and Timing	Patient Selection	Index Test	Reference Standard
Abuoglu 2016	+	+	+	+	+	+	+
Agarwal 2005	−	+	−	−	−	+	−
Ahadi 2017	+	?	−	+	+	?	−
Aihara 2004	+	?	?	+	+	?	?
Ali 2008	+	?	−	+	+	?	−
Al-Shibli 2005	+	+	?	+	+	+	?
Arlicot 2013	+	+	−	+	+	+	−
Arora 2007	+	+	?	+	+	+	?
Ballal 2017	+	+	+	−	+	+	+
Ballehaninna 2013	−	+	?	+	−	+	?
Barakat 2012	+	+	?	+	+	+	?
Bravo 2017	−	+	?	+	?	?	?
Brogi 2005	+	+	?	?	+	+	?
Celebioglu 2006	?	?	?	+	?	?	?
Chan 2011	+	+	?	−	+	+	?
Chao 2001	?	?	?	+	?	?	?
Choi 2006	?	?	?	+	?	?	?
Cipolla 2010	+	+	?	+	+	+	?
Cipolla 2020	?	+	?	+	?	+	?
Cotarelo 2020	+	+	?	+	+	+	?
Diest 1999	+	+	?	+	+	+	?
Elezoglu 2011	?	+	?	+	?	+	?
Flett 1998	?	?	?	+	?	?	?
Frere-Belda 2012	−	+	−	−	−	+	−
Geertsema 2010	−	+	?	+	−	+	?
Gemignani 2000	?	+	−	+	?	+	−
Gipponi 2004	+	+	?	+	+	+	?
Grabau 2005	−	+	−	−	−	+	−
Grabenster 2019	+	+	−	+	+	+	−
Han 2013	+	+	?	+	+	+	?
Hashmi 2013	+	+	−	+	+	+	−
Henry-Tillman 2002	?	?	?	+	?	?	?
Hill 1998	+	+	?	+	+	+	?
Hino 2008	+	+	?	−	+	+	?
Holck 2004	+	+	?	+	+	+	?
Horvath 2009	?	+	?	+	?	+	?
Houpu 2019	?	+	?	+	?	+	?
Hung 2005	+	?	?	−	+	?	?
Imoto 2000	?	?	?	+	?	?	?
Jaka 2010	+	+	?	+	+	+	?
Jamal 2011	+	+	?	+	+	+	?
Jara-Lazaro 2014	+	+	+	+	+	+	+
Jylling 2008	?	−	−	+	?	−	−
Kelley 1999	+	+	+	?	+	+	+
Khalifa 2004	+	?	?	+	+	?	?
Krishnamurthy 2009	?	?	?	+	?	?	?
Krogerus 2004	?	+	?	−	?	+	?
Lai 2018	−	?	?	−	−	?	?
Langer 2009	+	+	?	+	+	+	?
Lauridsen 2004	?	?	?	+	?	?	?
Lee 2006	+	+	?	−	+	+	?
Leidenius 2003	+	?	+	−	+	?	+
Leung 2007	+	−	?	+	+	−	?
Liang 2003	+	−	−	+	+	−	−
Lim 2013	+	+	−	+	+	+	−
Liu 2000	?	?	?	+	?	?	?
Liu 2011	+	+	?	+	+	+	?
Lombardi 2018	−	+	−	−	−	+	−
Lu 2013	−	+	?	−	−	+	?
Lumachi 2011	+	+	?	+	+	+	?
Lumachi 2012	+	+	?	+	+	+	?
McLaughlin 2008	−	+	?	−	−	+	?
Memar 2010	?	+	?	+	?	+	?
Menes 2003	?	+	?	+	?	+	?
Mitchell 2005	?	+	?	+	?	+	?
Moatasim 2013	?	?	+	+	?	?	+
Morgan 1999	?	?	?	+	?	?	?
Mori 2006	?	+	?	+	?	+	?
Motomura 2000	−	+	?	+	−	+	?
Nagashima 2003	?	+	?	+	?	+	?
Nahrig 2003	?	?	?	−	?	?	?
Nofech-Mozes 2009	?	+	?	+	?	+	?
Noguchi 2000	?	+	?	+	?	+	?
Nowikiewicz 2015	?	+	?	−	?	+	?
Perez 2005	?	+	+	+	?	+	+
Petropoulou 2017	+	+	+	+	+	+	+
Poling 2014	?	+	?	+	?	+	?
Qiao 2016	?	+	?	+	?	+	?
Rahusen 2000	+	+	?	−	+	+	?
Reitsamer 2004	+	+	?	−	+	+	?
Rubio 2010	?	+	?	−	?	+	?
Russo 2017	?	+	?	+	?	+	?
Safai 2012	?	?	?	+	?	?	?
Schrenk 2005	?	+	?	−	?	+	?
Schwartz 2008	?	+	?	+	?	+	?
Shimazu 2008	?	+	−	+	?	+	−
Shojaee 2020	?	+	−	+	?	+	−
Soares 2007	+	+	−	+	+	+	−
Somasherhs 2013	+	+	+	+	+	+	+
Stovagraad 2012	?	?	?	+	?	?	?
Sun 2017	+	+	+	+	+	+	+
Taffurelli 2012	+	+	−	+	+	+	−
Tan 2016	?	+	?	+	?	+	?
Tanis 2001	+	+	?	−	+	+	?
Tille 2009	?	?	?	+	?	?	?
Turner 1999	?	+	?	+	?	+	?
Upender 2009	+	+	?	+	+	+	?
Van der veen 2008	−	+	?	−	−	+	?
Veronesi 1997	?	+	?	+	?	+	?
Vohra 2015	+	+	?	+	+	+	?
Vrande 2008	−	+	?	−	−	+	?
Wada 2004	+	+	?	+	+	+	?
Wang 2012	?	+	?	+	?	+	?
Wang 2013	+	?	?	+	+	?	?
Weiser 2000	?	+	−	+	?	+	−
Wong 2014	?	+	−	+	?	+	−
Wong 2018	+	+	?	+	+	+	?
Yang 2000	?	+	?	+	?	+	?
Yoon 2019	?	+	?	+	?	+	?
Zurrida 2001	+	+	−	+	+	+	−

− High ? Unclear + Low
