# Supplementary figures and images for: The diagnostic accuracy of intraoperative frozen section biopsy for diagnosis of sentinel lymph node metastasis in breast cancer patients: a meta-analysis
Source: Environ Sci Pollut Res Int. 2022 May 11;29(32):47931–41. doi: 10.1007/s11356-022-20569-4 (PMC9252966; doi:10.1007/s11356-022-20569-4)

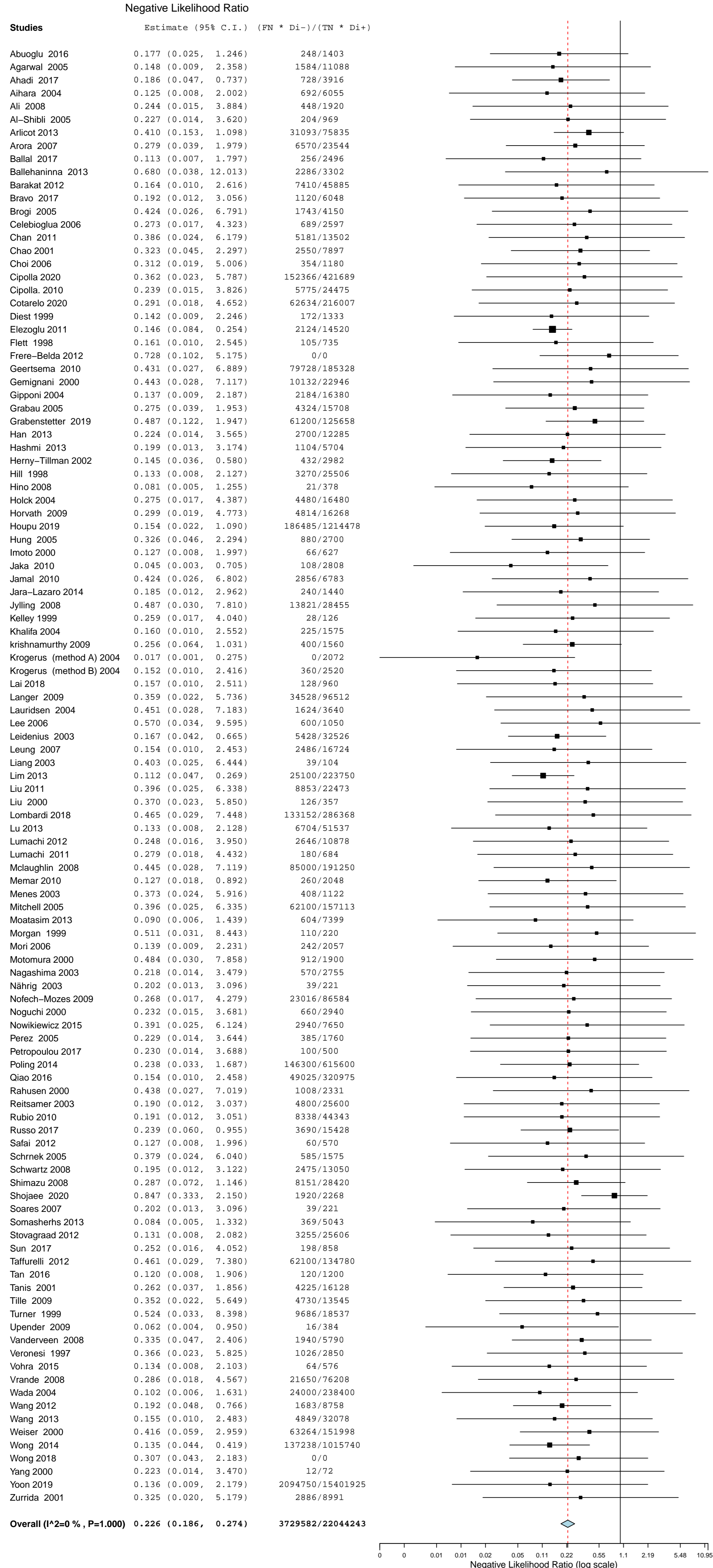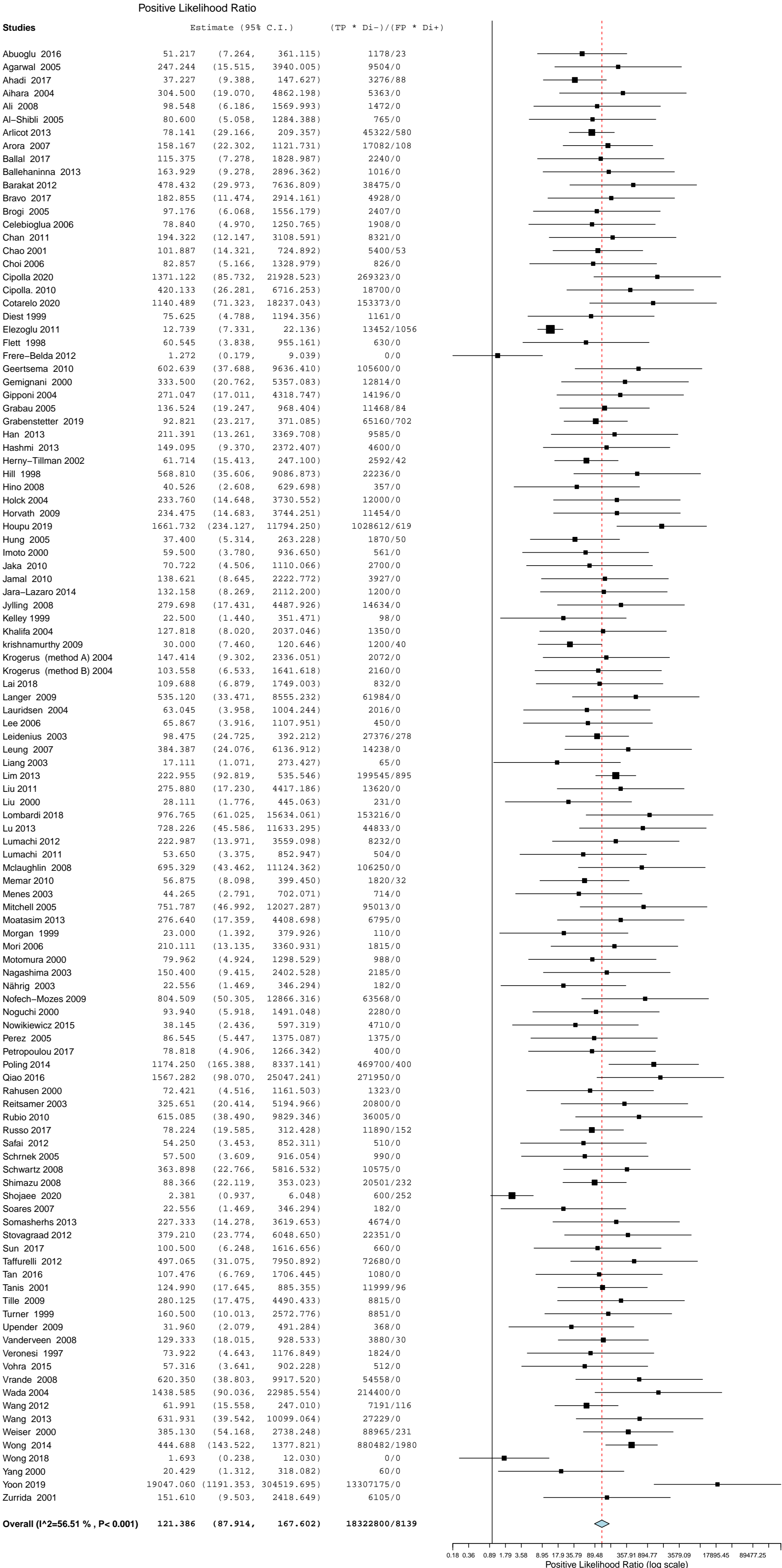

Supplement: Supplementary file 2 — Supplementary Fig. 2: Forest plots for the pooled negative likelihood ratio (to the left) and positive likelihood ratio (to the right) of intraoperative frozen section biopsy in detecting sentinel lymph node metastasis in breast cancer patients. (PDF 19 KB) [file 11356_2022_20569_MOESM2_ESM.pdf]
