# Supplementary material for: The diagnostic accuracy of intraoperative frozen section biopsy for diagnosis of sentinel lymph node metastasis in breast cancer patients: a meta-analysis
Source: Environ Sci Pollut Res Int. 2022 May 11;29(32):47931–41. doi: 10.1007/s11356-022-20569-4 (PMC9252966; doi:10.1007/s11356-022-20569-4)

Studies	Estimate (95% C.I.)	(TP * TN)/(FP * FN)
Abuoglu 2016	289.750 (30.509, 2751.809)	1159/4
Agarwal 2005	1671.174 (97.028, 28783.608)	9504/0
Ahadi 2017	200.250 (40.547, 988.989)	3204/16
Aihara 2004	2429.000 (127.605, 46236.839)	5363/0
Ali 2008	404.200 (22.209, 7356.299)	1472/0
Al−Shibli 2005	354.778 (18.085, 6959.753)	765/0
Arlicot 2013	190.585 (67.497, 538.140)	44978/236
Arora 2007	566.800 (76.015, 4226.293)	17004/30
Ballal 2017	1017.667 (53.248, 19449.566)	2240/0
Ballehaninna 2013	241.105 (12.092, 4807.329)	1016/0
Barakat 2012	2919.642 (176.606, 48267.406)	38475/0
Bravo 2017	953.571 (54.709, 16620.679)	4928/0
Brogi 2005	229.140 (13.453, 3902.744)	2407/0
Celebioglua 2006	289.296 (16.670, 5020.635)	1908/0
Chan 2011	503.060 (30.299, 8352.333)	8321/0
Chao 2001	315.529 (40.645, 2449.457)	5364/17
Choi 2006	265.462 (14.130, 4987.178)	826/0
Cipolla 2020	3789.267 (235.067, 61082.871)	269323/0
Cipolla. 2010	1755.512 (105.026, 29343.538)	18700/0
Cotarelo 2020	3920.261 (241.974, 63512.866)	153373/0
Diest 1999	531.667 (27.537, 10265.243)	1161/0
Elezoglu 2011	87.083 (37.406, 202.733)	12540/144
Flett 1998	375.286 (18.388, 7659.489)	630/0
Frere−Belda 2012	1.747 (0.035, 88.393)	0/0
Geertsema 2010	1398.868 (86.685, 22573.948)	105600/0
Gemignani 2000	752.739 (45.324, 12501.474)	12814/0
Gipponi 2004	1975.138 (116.446, 33502.009)	14196/0
Grabau 2005	495.957 (65.603, 3749.420)	11407/23
Grabenstetter 2019	190.582 (46.742, 777.070)	64798/340
Han 2013	945.195 (56.339, 15857.349)	9585/0
Hashmi 2013	747.400 (43.344, 12887.666)	4600/0
Herny−Tillman 2002	426.000 (66.637, 2723.346)	2556/6
Hill 1998	4273.095 (247.431, 73795.643)	22236/0
Hino 2008	501.667 (19.215, 13097.219)	357/0
Holck 2004	850.368 (51.229, 14115.555)	12000/0
Horvath 2009	784.525 (47.271, 13020.197)	11454/0
Houpu 2019	10821.979 (1505.268, 77803.594)	1028088/95
Hung 2005	114.750 (14.547, 905.155)	1836/16
Imoto 2000	469.000 (21.321, 10316.565)	561/0
Jaka 2010	1574.714 (79.235, 31295.960)	2700/0
Jamal 2010	326.796 (19.362, 5515.847)	3927/0
Jara−Lazaro 2014	713.000 (35.052, 14503.235)	1200/0
Jylling 2008	574.631 (34.930, 9453.223)	14634/0
Kelley 1999	87.000 (3.685, 2054.221)	98/0
Khalifa 2004	798.143 (39.480, 16135.567)	1350/0
krishnamurthy 2009	117.000 (20.733, 660.237)	1170/10
Krogerus (method A) 2004	8493.000 (164.576, 438284.550)	2072/0
Krogerus (method B) 2004	679.462 (37.176, 12418.280)	2160/0
Lai 2018	696.600 (31.617, 15347.690)	832/0
Langer 2009	1491.419 (91.938, 24193.772)	61984/0
Lauridsen 2004	139.814 (8.284, 2359.788)	2016/0
Lee 2006	115.471 (5.968, 2234.047)	450/0
Leidenius 2003	590.087 (136.773, 2545.836)	27144/46
Leung 2007	2501.348 (145.404, 43030.016)	14238/0
Liang 2003	42.429 (1.865, 965.338)	65/0
Lim 2013	1987.500 (735.723, 5369.083)	198750/100
Liu 2011	696.899 (42.223, 11502.359)	13620/0
Liu 2000	76.077 (3.926, 1474.345)	231/0
Lombardi 2018	2099.061 (130.041, 33881.980)	153216/0
Lu 2013	5466.212 (325.344, 91839.626)	44833/0
Lumachi 2012	900.946 (53.396, 15201.485)	8232/0
Lumachi 2011	192.455 (9.990, 3707.674)	504/0
Mclaughlin 2008	1562.604 (96.744, 25239.023)	106250/0
Memar 2010	448.000 (47.892, 4190.800)	1792/4
Menes 2003	118.680 (6.678, 2109.010)	714/0
Mitchell 2005	1898.512 (117.275, 30734.152)	95013/0
Moatasim 2013	3063.667 (161.887, 57979.044)	6795/0
Morgan 1999	45.000 (2.149, 942.120)	110/0
Mori 2006	1506.600 (69.101, 32848.064)	1815/0
Motomura 2000	165.240 (9.227, 2959.297)	988/0
Nagashima 2003	690.538 (37.556, 12696.783)	2185/0
Nährig 2003	111.857 (5.274, 2372.550)	182/0
Nofech−Mozes 2009	3007.071 (183.735, 49214.648)	63568/0
Noguchi 2000	405.087 (23.196, 7074.217)	2280/0
Nowikiewicz 2015	97.538 (5.897, 1613.235)	4710/0
Perez 2005	377.400 (20.748, 6864.805)	1375/0
Petropoulou 2017	343.400 (15.132, 7793.112)	400/0
Poling 2014	4941.000 (686.252, 35575.113)	469395/95
Qiao 2016	10189.150 (627.199, 165527.756)	271950/0
Rahusen 2000	165.485 (9.518, 2877.158)	1323/0
Reitsamer 2003	1710.388 (102.982, 28407.033)	20800/0
Rubio 2010	3221.533 (193.680, 53584.622)	36005/0
Russo 2017	327.056 (73.729, 1450.787)	11774/36
Safai 2012	427.000 (19.376, 9409.841)	510/0
Schrnek 2005	151.667 (8.621, 2668.303)	990/0
Schwartz 2008	1862.826 (107.898, 32161.017)	10575/0
Shimazu 2008	308.106 (72.357, 1311.951)	20335/66
Shojaee 2020	2.812 (0.934, 8.471)	540/192
Soares 2007	111.857 (5.274, 2372.550)	182/0
Somasherhs 2013	2717.000 (137.290, 53770.151)	4674/0
Stovagraad 2012	2904.677 (172.123, 49018.146)	22351/0
Sun 2017	399.000 (19.204, 8289.848)	660/0
Taffurelli 2012	1077.332 (66.651, 17413.825)	72680/0
Tan 2016	895.400 (41.124, 19495.895)	1080/0
Tanis 2001	477.120 (63.423, 3589.290)	11928/25
Tille 2009	794.956 (47.286, 13364.394)	8815/0
Turner 1999	306.368 (18.619, 5041.094)	8851/0
Upender 2009	517.000 (19.809, 13493.597)	368/0
Vanderveen 2008	386.000 (46.959, 3172.877)	3860/10
Veronesi 1997	202.027 (11.783, 3463.921)	1824/0
Vohra 2015	429.000 (19.450, 9462.405)	512/0
Vrande 2008	2171.792 (133.057, 35448.505)	54558/0
Wada 2004	14094.049 (858.903, 231274.235)	214400/0
Wang 2012	322.591 (69.033, 1507.455)	7097/22
Wang 2013	4067.000 (239.112, 69174.660)	27229/0
Weiser 2000	925.313 (127.907, 6693.963)	88830/96
Wong 2014	3291.270 (1037.457, 10441.355)	878769/267
Wong 2018	5.510 (0.107, 284.034)	0/0
Yang 2000	91.667 (3.203, 2623.355)	60/0
Yoon 2019	139772.087 (8705.322, 2244171.674)	13307175/0
Zurrida 2001	467.038 (27.941, 7806.486)	6105/0
**Overall (I^2=53.96 % , P< 0.001)**	**569.537 (404.236, 802.434)**	**18316557/1896**

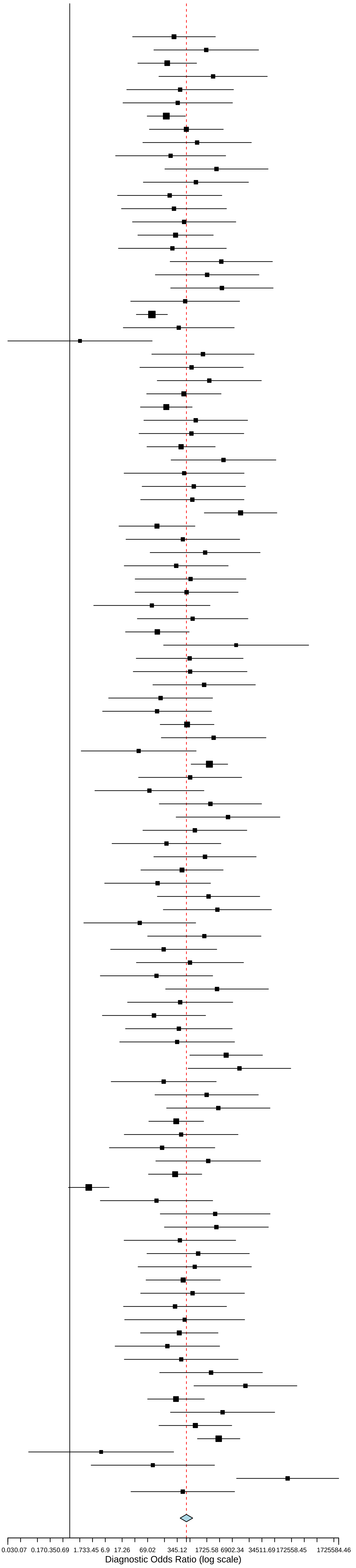

Supplement: Supplementary file 3 — Supplementary Fig. 3: A forest blot for the pooled diagnostic odds ratio of intraoperative frozen section biopsy in the detection of sentinel lymph node metastasis in breast cancer patients. (PDF 12 KB) [file 11356_2022_20569_MOESM3_ESM.pdf]
