# Supplementary material for: The diagnostic accuracy of intraoperative frozen section biopsy for diagnosis of sentinel lymph node metastasis in breast cancer patients: a meta-analysis
Source: Environ Sci Pollut Res Int. 2022 May 11;29(32):47931–41. doi: 10.1007/s11356-022-20569-4 (PMC9252966; doi:10.1007/s11356-022-20569-4)

| Studies | Estimate (95% C.I.) | | | (TP * TN)/(FP * FN) |
|---|---|---|---|---|
| Barakat 2012 | 1.044 | (0.020, | 54.916) | 0/0 |
| Brogi 2005 | 0.268 | (0.005, | 15.115) | 0/0 |
| Celebioglua 2006 | 0.556 | (0.010, | 30.953) | 0/0 |
| Chan 2011 | 208.484 | (11.708, | 3712.528) | 1540/0 |
| Choi 2006 | 5.000 | (0.150, | 166.589) | 4/0 |
| Cipolla 2020 | 0.132 | (0.003, | 6.967) | 0/0 |
| Cotarelo 2020 | 247.198 | (13.463, | 4538.890) | 5110/0 |
| Grabau 2005 | 0.415 | (0.008, | 22.653) | 0/0 |
| Hashmi 2013 | 0.520 | (0.009, | 29.340) | 0/0 |
| Houpu 2019 | 0.131 | (0.005, | 3.387) | 0/39 |
| Krogerus (method A) 2004 | 15.000 | (0.117, | 1923.713) | 0/0 |
| Langer 2009 | 0.122 | (0.002, | 6.609) | 0/0 |
| Lauridsen 2004 | 0.378 | (0.007, | 20.596) | 0/0 |
| Leidenius 2003 | 1.629 | (0.031, | 85.847) | 0/0 |
| Leung 2007 | 0.733 | (0.014, | 38.897) | 0/0 |
| Lim 2013 | 489.300 | (54.964, | 4355.826) | 4893/10 |
| Lombardi 2018 | 0.233 | (0.005, | 12.069) | 0/0 |
| Menes 2003 | 0.294 | (0.005, | 18.955) | 0/0 |
| Nagashima 2003 | 1.000 | (0.016, | 62.300) | 0/0 |
| Nährig 2003 | 1.800 | (0.027, | 121.703) | 0/0 |
| Nofech−Mozes 2009 | 0.333 | (0.006, | 18.885) | 0/0 |
| Nowikiewicz 2015 | 0.119 | (0.002, | 6.534) | 0/0 |
| Perez 2005 | 1.667 | (0.020, | 137.354) | 0/0 |
| Rahusen 2000 | 0.407 | (0.007, | 23.230) | 0/0 |
| Russo 2017 | 0.524 | (0.009, | 30.168) | 0/0 |
| Shimazu 2008 | 0.467 | (0.009, | 25.170) | 0/0 |
| Stovagraad 2012 | 0.220 | (0.004, | 12.162) | 0/0 |
| Taffurelli 2012 | 0.390 | (0.008, | 20.150) | 0/0 |
| Tille 2009 | 0.692 | (0.011, | 41.777) | 0/0 |
| Turner 1999 | 0.130 | (0.002, | 7.073) | 0/0 |
| Vrande 2008 | 1436.841 | (86.836, | 23774.777) | 22425/0 |
| Wada 2004 | 0.333 | (0.006, | 18.885) | 0/0 |
| Weiser 2000 | 0.326 | (0.006, | 16.792) | 0/0 |
| Wong 2014 | 1.000 | (0.019, | 51.887) | 0/0 |
| **Overall (I^2=62.56 % , P< 0.001)** | **1.347** | **(0.468,** | **3.882)** | **33972/49** |

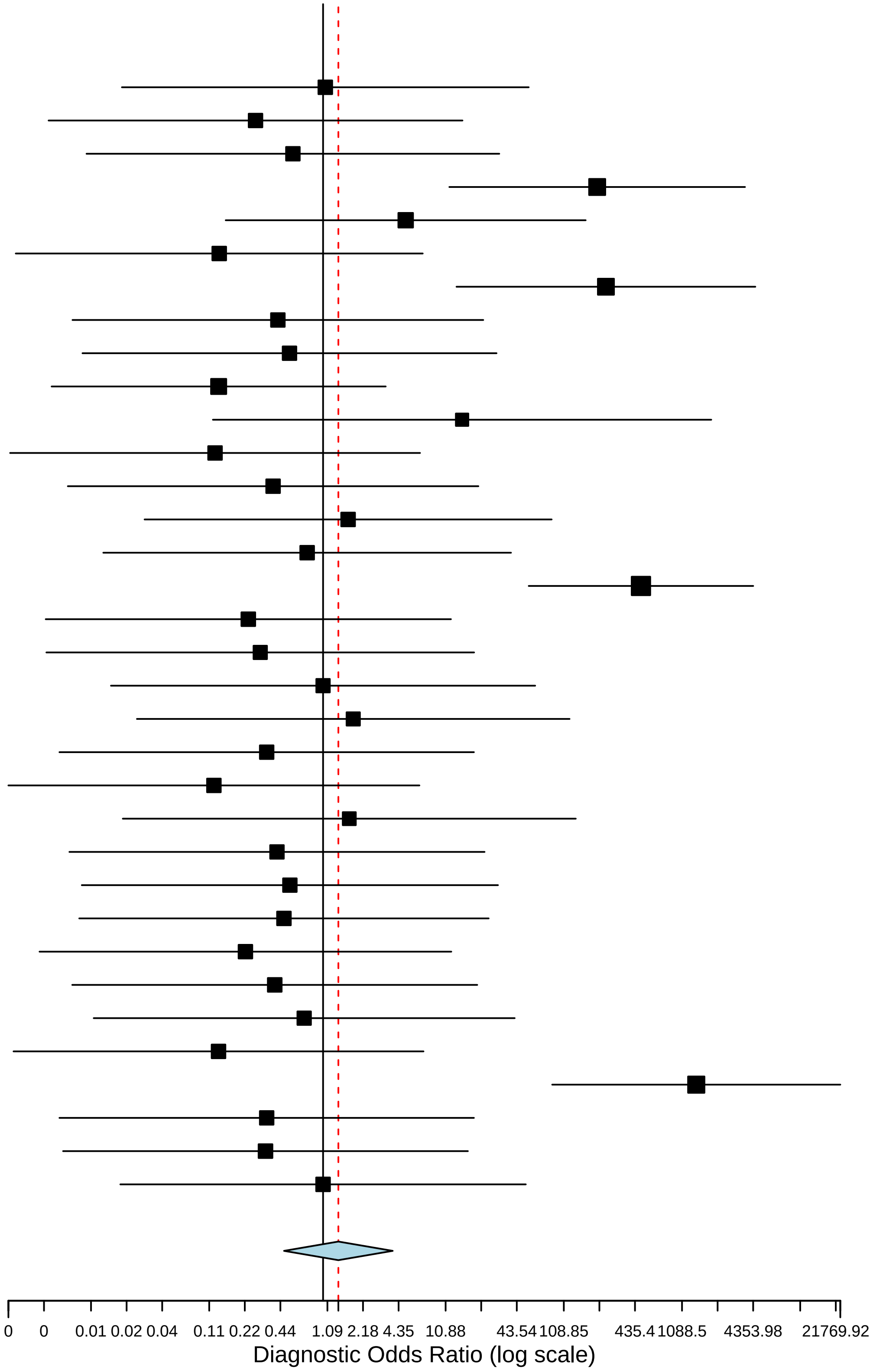

Supplement: Supplementary file 4 — Supplementary Fig. 4: A forest blot for the pooled diagnostic odds ratio of intraoperative frozen section biopsy in the detection of sentinel lymph node micro-metastasis in breast cancer patients. (PDF 7 KB) [file 11356_2022_20569_MOESM4_ESM.pdf]
