# Supplementary material for: The diagnostic accuracy of intraoperative frozen section biopsy for diagnosis of sentinel lymph node metastasis in breast cancer patients: a meta-analysis
Source: Environ Sci Pollut Res Int. 2022 May 11;29(32):47931–41. doi: 10.1007/s11356-022-20569-4 (PMC9252966; doi:10.1007/s11356-022-20569-4)

Studies	Estimate	(95% C.I.)		(TP * TN)/(FP * FN)
Barakat 2012	25.000	(0.444,	1408.747)	0/0
Brogi 2005	16.333	(0.232,	1147.900)	0/0
Celebioglua 2006	55.000	(0.446,	6787.046)	0/0
Chan 2011	2849.667	(113.108,	71795.186)	2091/0
Choi 2006	25.000	(0.199,	3139.088)	0/0
Cipolla 2020	4.938	(0.097,	251.904)	0/0
Cotarelo 2020	9503.149	(579.678,	155793.255)	158658/0
Grabau 2005	15.286	(0.262,	891.823)	0/0
Hashmi 2013	89.000	(0.725,	10921.953)	0/0
Houpu 2019	13.453	(0.263,	687.573)	0/0
Krogerus (method A) 2004	43.000	(0.347,	5327.749)	0/0
Langer 2009	41.571	(0.717,	2411.915)	0/0
Lauridsen 2004	3.800	(0.069,	207.806)	0/0
Leidenius 2003	13.615	(0.249,	743.397)	0/0
Leung 2007	0.905	(0.018,	46.500)	0/0
Lim 2013	2093.800	(647.672,	6768.860)	83752/40
Lombardi 2018	2.960	(0.058,	151.139)	0/0
Menes 2003	4.333	(0.075,	249.152)	0/0
Nagashima 2003	43.000	(0.347,	5327.749)	0/0
Nährig 2003	11.000	(0.084,	1437.995)	0/0
Nowikiewicz 2015	3.092	(0.061,	157.676)	0/0
Perez 2005	47.000	(0.380,	5814.172)	0/0
Rahusen 2000	4.714	(0.079,	280.535)	0/0
Russo 2017	6.294	(0.117,	338.928)	0/0
Shimazu 2008	10.273	(0.185,	570.203)	0/0
Stovagraad 2012	9.769	(0.179,	534.538)	0/0
Taffurelli 2012	7.848	(0.151,	408.948)	0/0
Turner 1999	31.000	(0.445,	2159.729)	0/0
Vrande 2008	558.231	(33.078,	9420.896)	5368/0
Wada 2004	6.429	(0.121,	341.805)	0/0
Weiser 2000	10.579	(0.198,	563.883)	0/0
Wong 2014	30.429	(0.587,	1578.332)	0/0
**Overall (I^2=60.06 % , P< 0.001)**	**29.245**	**(10.290,**	**83.114)**	**249869/40**

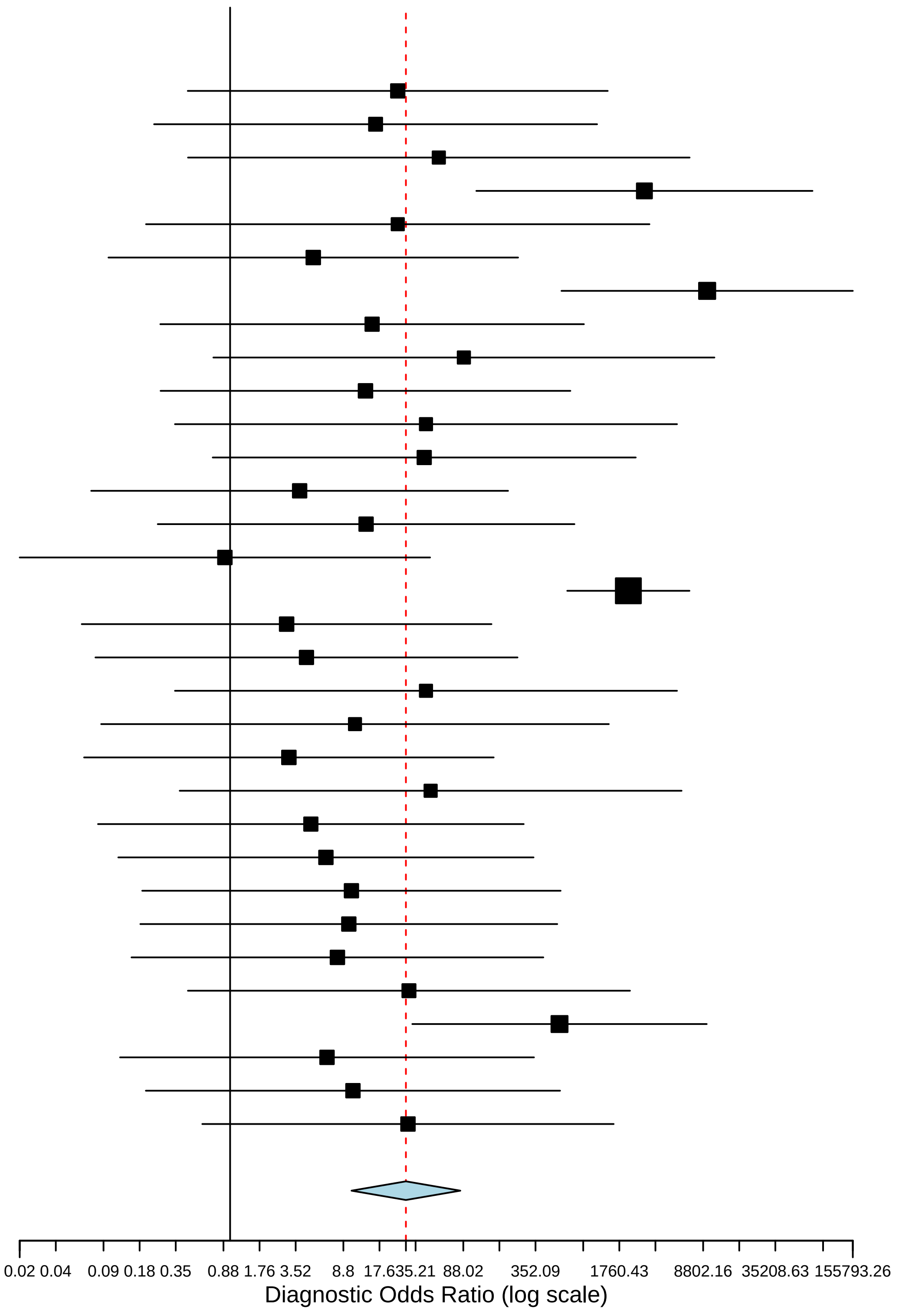

Supplement: Supplementary file 5 — Supplementary Fig. 5: A forest blot for the pooled diagnostic odds ratio of intraoperative frozen section biopsy in detecting sentinel lymph node macro-metastasis in breast cancer patients. (PDF 7 KB) [file 11356_2022_20569_MOESM5_ESM.pdf]
