# Supplementary material for: The diagnostic accuracy of intraoperative frozen section biopsy for diagnosis of sentinel lymph node metastasis in breast cancer patients: a meta-analysis
Source: Environ Sci Pollut Res Int. 2022 May 11;29(32):47931–41. doi: 10.1007/s11356-022-20569-4 (PMC9252966; doi:10.1007/s11356-022-20569-4)
Supplement: Supplementary file 6 — Supplementary file6 (DOCX 263 KB) [file 11356_2022_20569_MOESM6_ESM.docx]

**Supplementary table 1: Summary of the included studies.^1-110^**

| Study ID | Design | Total No. of patients | Total No. of SLN | Comparator | Conclusion |
| --- | --- | --- | --- | --- | --- |
| Abuoglu 2016 | Retrospective | 85 | 85 | Paraffin Block | FS is a good method in evaluation non palpable masses with sensitivity over 80% and accuracy over 90%. |
| Agarwal 2005 | Prospective | 234 | 221 | Paraffin Block | FS prevents second operation in 80% of patients with positive SLN. |
| Ahadi 2017 | Retrospective | 135 | 135 | Permanent section | IFS is a helpful method to evaluate SLN in breast cancer patients, that can prevent second operation and help in determination of immediate axillary dissection. |
| Aihara 2004 | Prospective | 107 | 208 | Paraffin sections | FS and TIC almost have the same sensitivity. However, TIHC is more sensitive than both. |
| Ali 2008 | Prospective | 94 | 94 | Paraffin sections | FS is a reliable method that enables most cases to avoid second surgery. |
| Al-Shibli 2005 | Retrospective | 70 | 139 | Paraffin section | FS for SLN evaluation is 100% specific and 79% sensitive. However, diagnosis of small tumors (lobular carcinoma) may need IHC with cytokeratin. Diagnosis of small metastasis by FS may be challenging. TIC adds nothing to the diagnosis. |
| Arlicot 2013 | Retrospective | 672 | 2341 | Definitive histopathology | FS failed in detection of micrometastasis as well as macrometastasis. |
| Arora 2007 | Retrospective | 659 | 578 | Permanent section | FS diagnosed small metastases and didn't produce a high FN rate. IFS provides immediate ALND. |
| Ballal 2017 | Retrospective | 104 | 104 | Permanent sections | FS is highly sensitive in macrometastasis detection in early stages of breast cancer which in turn reduces the need for second operation. |
| Ballehaninna 2013 | Retrospective | 267 | 563 | Paraffin sections | IFS has a low yield sensitivity for SLN evaluation in DCIS. |
| Barakat 2012 | Retrospective | 440 | 449 | Paraffin sections | FS is a reliable method for SLN evaluation. |
| Espinosa-Bravo 2017 | Prospective | 320 | 689 | Paraffin blocks | Sensitivity of FS was 81.5% due to failure in detection of micrometastasis |
| Brogi 2005 | Prospective | 133 | 305 | Paraffin sections | FS is more sensitive in detection macrometastasis than micrometastasis |
| Celebioglua 2006 | Prospective | 102 | 1.8 per case | Paraffin sections | Combination of H&E and IHC did raise the sensitivity in detecting macrometastases but not micrometastases. |
| Chan 2011 | Prospective | 325 | 329 | Paraffin sections | FS failed in detection of micrometastasis. However, it was helpful in macrometastasis detection hence prevented second operation. |
| Chao 2001 | Retrospective | 200 | 203 | Paraffin sections | FS spared two thirds of patients a second surgery. |
| Choi 2006 (IHC) | Prospective | 79 | 178 | Paraffin sections | Ultrarapid immunohistochemical method is more sensitive than FS stained with H&E. |
| Cipolla 2010 | Retrospective | 364 | 368 | Definitive histopathology | IFS is a reliable method for axillary lymph node evaluation in breast cancer. It allows one-stage operation. |
| Cipolla 2020 | Retrospective | 1456 | 1470 | Paraffin sections | FS is a very useful method for evaluation of SLN that prevents a second operation in node-positive patients. |
| Cotarelo 2020 | Retrospective | 1072 | 2276 | Paraffin sections | FS can spare patients with extended axillary involvement a second surgery. However, it isn't useful in patients with no SLN metastasis. |
| Van Diest 1999 | Prospective | 54 | 74 | Paraffin sections | FS is a reliable method that allows patients with positive SLN to have immediate axillary lymph node dissection. |
| Elezoglu 2011 | Retrospective | 265 | 265 | Permanent sections. | FS failed to detect 2.2% of metastasis that was reported to be micrometastasis on permanent sections. |
| Flett 1998 | Prospective | 68 | 69 | Paraffin section | This technique allowed selective policy in only one patient. |
| Le Frere-Belda 2012 | Prospective | 233 | 503 | Paraffin sections | One-step nucleic acid amplification assay (OSNA) has a higher sensitivity than intraoperative histological evaluation. OSNA is predicted to decrease number of patients undergoing second operation for ALND. |
| Geertsema 2010 | Prospective | 1004 | 1004 | Paraffin sections | FS spares patients with positive SLNs a second operation. |

SLN: sentinel lymph node; FS: frozen section; IFS: intraoperative frozen section; TIC: touch imprint cytology; TIHC: touch imprint cytology with immunohistochemical staining; FN: false negative; FNR: false negative rate; NAC: neoadjuvant chemotherapy; ITCs: isolated tumor cells; ALND: axillary lymph node dissection.

**Supplementary table 1, continues: Summary of the included studies.**

| Study ID | Design | Total No. of patients | Total No. of SLN | Comparator | Conclusion |
| --- | --- | --- | --- | --- | --- |
| Gemignani 2000 | Retrospective | 432 | 375 | Permanent section | 10% of patients with T1 tumors evaluated by FS will have a second operation. |
| Gipponi 2004 | Prospective | 334 | 326 | Definitive Histology | FS predicted the actual status of SLN and allowed a one-stage operation in 35% of patients. |
| Grabau 2005 | Prospective | 352 | 272 | Paraffin sections | FS failed to detect all micrometastasis with 71% (20/28) false negative rate. |
| Grabenstetter 2019 | Prospective | 702 | 711 | Paraffin sections | Errors during SLN sampling are common, they resulted in (25/28) of FN cases. |
| Han 2013 | Retrospective | 226 | 263 | Paraffin sections | FS examination results after NAC and without NAC are similar in patients with early breast cancer stages. |
| Hashmi 2013 | Retrospective | 154 | 154 | Paraffin sections | FS is unreliable in detection of micrometastasis. |
| Herny-Tillman 2002 | Retrospective | 255 | 479 | Paraffin sections | Touch preparation cytology is simpler and faster than FS with the same accuracy. |
| Hill 1998 | Prospective | 500 | 458 | Paraffin section | Intraoperative evaluation of SLN by FS is safe and reliable to avoid ALND in node-negative patients. Due to FN rate, this method is most effective in patients at low risk of axillary nodal metastases. |
| Hino 2008 | Prospective | 55 | 66 | Permanent sections | SLNB was an appropriate and accurate method to predict the axillary lymph node status even in patients with operable breast cancer after NAC. |
| Holck 2004 | Prospective | 265 | 265 | Paraffin sections | FS is a reliable method to detect macrometastasis and spares most patients a second operation but it failed in detection of micrometastasis. |
| Horvath 2009 | Retrospective | 1621 | 264 | Permanent section | FS results in patients with invasive lobular carcinoma should be considerable to reduce the need of a second operation. |
| Houpu 2019 | Retrospective | 2552 | 2582 | Paraffin section | FNR of FS was significantly bigger in micrometastasis than macrometastasis. |
| Hung 2005 | Prospective | 123 | 105 | Paraffin sections | IFS wasn't accurate enough as FN rate was 32%. |
| Imoto 2000 | Prospective | 59 | 2.5 per case | Paraffin sections | FS identified SLN in 52 cases with 89% sensitivity and 96% accuracy. |
| Jaka 2010 | Retrospective | 114 | 114 | Paraffin section | FS is highly accurate in SLN metastasis detection even in previously treated patients. |
| Jamal 2011 | Retrospective | 176 | 376 | Paraffin section | Negative SLN detected by FS is not likely to have additional metastasis. |
| Jara-Lazaro 2014 | Prospective | 55 | 100 | Paraffin section | One step nucleic acid amplification (OSNA) has similar accuracy as FS or even better detection for micrometastasis, |
| Jylling 2008 | Prospective | 456 | 872 | Paraffin section | FS spared 71% of patients a second operation. |
| Kelley 1999 | Retrospective | 28 | 23 | Permanent section | Studies evaluating SLN examination must take surgical pathology techniques into account, as different techniques yield different numbers of positive sentinel nodes. |
| Khalifa 2004 | Prospective | 96 | 196 | Paraffin sections | FS is highly accurate for macrometastases but not for micrometastases |
| Krishnamurthy 2009 | Prospective | 100 | 297 | Paraffin sections | FS detected all (12/12) of macrometastases but only (3/8) of micrometatases. |
| Krogerus 2004 | Prospective | 204 | 204 | Paraffin sections | Method A failed to detect small metastases detected by method B in addition method B takes less time and effort than method A. |
| Lai 2018 | Retrospective | 82 | 77 | Paraffin sections | IFS is a reliable method in SLN evaluation. |
| Langer 2009 | Prospective | 698 | 648 | Paraffin sections | FS is highly accurate in macrometastases detection and can prevent second operation in most patients. |
| Lauridsen 2004 | Prospective | 124 | 121 | Paraffin section | This method has proven its reliability in sparing node-negative patients a second surgery. |
| Lee 2006 | Prospective | 356 | 89 | Paraffin sections | FS provides better information but due to cost-effectiveness, tissue loss and time consumption, Rapid cytokeratin immunochemistry (CK-IHC) should be applied. |
| Leidenius 2003 | Retrospective | 375 | 139 | Paraffin sections | FS allows treatment, axillary staging, and surgery in the same operation. |

SLN: sentinel lymph node; FS: frozen section; IFS: intraoperative frozen section; TIC: touch imprint cytology; TIHC: touch imprint cytology with immunohistochemical staining; FN: false negative; FNR: false negative rate; NAC: neoadjuvant chemotherapy; ITCs: isolated tumor cells; ALND: axillary lymph node dissection.

**Supplementary table 1, continues: Summary of the included studies.**

| Study ID | Design | Total No. of patients | Total No. of SLN | Comparator | Conclusion |
| --- | --- | --- | --- | --- | --- |
| Leung 2007 | Retrospective | 300 | 1172 | Paraffin sections | IFS is a reliable method for SLN evaluation. 85% of patients with positive metastases avoided second operation. FS is less accurate in patients with small tumors |
| Liang 2003 | Prospective | 20 | 21 | Paraffin sections | FS and TIC have similar accuracy in SLN evaluation. |
| Lim 2013 | Retrospective | 717 | 1129 | Paraffin blocks |  |
| Liu 2000 | Prospective | 41 | 1.5 per case | Permanent section | FS enables further improvement in detecting micrometastases SLN in breast cancer patients. |
| Liu 2011 | Retrospective | 326 | 3.2 per patient | Permanent sections | FS is more accurate in detection of macrometastases than micrometastases. |
| Lombardi 2018 | Retrospective | 1453 | 1430 | Paraffin section | FS has low sensitivity in micrometastases detection but a rational sensitivity in macromeatstases |
| Lu 2013 | Retrospective | 586 | 574 | Permanent sections | IFS is a reliable method for SLN detection that allows axillary lymph node dissection in the same operation. |
| Lumachi 2011 | Prospective | 112 | 112 | Paraffin sections | FS failed to identify 7 cases diagnosed by final pathology |
| Lumachi 2012 | Prospective | 126 | 221 | Permanent histology | Combination of TIC and FS might be useful in SLN detection in early stages of breast cancer. |
| McLaughlin 2008 | Prospective | 2988 | 931 | Paraffin section | FS detected and allowed immediate axillary lymph node dissection for only 56% of patients with positive SLN while 44% FN patients needed a second operation. |
| Memar 2010 | Prospective | 97 | 147 | Permanent section | FS is a reliable method in order to avoid the second surgery. However, it can be saved for negative results of TIC. |
| Menes 2003 | Retrospective | 117 | 117 | Paraffin section | FS and touch preparation cytology are accurate for detection of macrometastases not micrometastases. Touch preparation method provides quick evaluation of SLN without wasting tissue. |
| Mitchell 2005 | Retrospective | 908 | 2.4 per case | Paraffin blocks | FS failed to detect sub-micrometastases tumor cell. |
| Moatasim 2013 | Prospective | 100 | 200 | Paraffin section | FS is a reliable and accurate method in immediate early detection of SLN at time of surgery. |
| Morgan 1999 | Prospective | 44 | 32 | Paraffin sections | |
| Mori 2006 | Prospective | 138 | 231 | Paraffin section | FS is better than TIC for intra-operative detection of SLN in patients with stage I breast cancer. |
| Motomura 2000 | Prospective | 101 | 158 | Paraffin section | TIC is better than FS and can detect micrometastases more accurately than routinely H&E sectioning. |
| Nagashima 2003 | Prospective | 124 | 303 | Paraffin section | FS and TIC should be combined to improve the diagnostic sensitivity. |
| Nährig 2003 | Prospective | 50 | 33 | Paraffin section | Ultrarapid IHC increased the sensitivity of FS evaluation of SLN by 5% so, it may be useful in cases cant't be detected by FS H&E. |
| Nofech-Mozes 2009 | Retrospective | 706 | 2.4 ± 1.6 per case | Paraffin section | Intraoperative consultation by FS is a safe reliable method that can prevent a second surgery in node- negative patients. |
| Noguchi 2000 | Prospective | 140 | 123 | Paraffin section | FS allowed to avoid axillary lymph node dissection in node-negative patients or patients with small breast cancers. |
| Nowikiewicz 2015 | Prospective | 1358 | 1285 | Paraffin blocks | Sensitivity of IFS in case of macrometastases was 75.9% while in micrometastases was 11.3%. |
| Perez 2005 | Prospective | 86 | 152 | Paraffin section | FS is a highly reliable, fast and cost-effective method in macrometastases detection. |

SLN: sentinel lymph node; FS: frozen section; IFS: intraoperative frozen section; TIC: touch imprint cytology; TIHC: touch imprint cytology with immunohistochemical staining; FN: false negative; FNR: false negative rate; NAC: neoadjuvant chemotherapy; ITCs: isolated tumor cells; ALND: axillary lymph node dissection.

**Supplementary table 1, continues: Summary of the included studies.**

| Study ID | Design | Total No. of patients | Total No. of SLN | Comparator | Conclusion |
| --- | --- | --- | --- | --- | --- |
| Petropoulou 2017 | Prospective | 60 | 80 | Paraffin section | TIC is likely to be more sensitive than FS for intra-operative evaluation of SLN in patients with breast cancer. |
| Poling 2014 | Retrospective | 1940 | 1940 | Paraffin section | FS shows excellent specificity when most of the FN is due to small tumors and tissue sampling. |
| Qiao 2016 | Retrospective | 1272 | 1272 | Paraffin section | FS is a useful method in SLN detection but not micrometastases. |
| Rahusen 2000 | Prospective | 115 | 1.6 per case | Paraffin sections | FS showed sensitivity in 57% of patients with Positive SLN while only (21/37) patients benefited from having axillary lymph node dissection in the same surgery. |
| Reitsamer 2003 | Prospective | 333 | 328 | Paraffin sections | FS failed to detect metastases in 24 patients found to be positive in permanent section, so they had second operation. |
| Rubio 2010 | Prospective | 498 | 496 | Paraffin section | FS is a sensitive method for macrometastases detection but it has lower sensitivity for micrometastases and sub-micrometastases. FS after NAC can help avoiding second surgery in node-positive patients. |
| Russo 2017 | Retrospective | 281 | 620 | Paraffin section | We believe that broad information of the definitive histopathological findings either of the primary tumor and SLNs may help in making a better decision for axillary lymph node dissection without FS. |
| Safai 2012 | Prospective | 49 | 100 | Permanent Histology | There's no difference between FS and permanent section results in SLNs evaluation. |
| Schrnek 2005 | Retrospective | 80 | 80 | Permanent Histology | Performing a SLN biopsy is recommended by the authors in order to avoid a secondary axillary lymph node dissection. |
| Schwartz 2008 | Retrospective | 283 | 283 | Paraffin section | Data verify the value of FS examination of SLN with sensitivity over 95%. FS failed to detect micrometastases in case of in situ ductal carcinoma or lymphovascular invasion |
| Shimazu 2008 | Retrospective | 398 | 363 | Paraffin section | No difference in IFS accuracy of SLN in patients treated with or without NAC. |
| Shojaee 2020 | Prospective | 102 | 102 | Definitive pathology | FN was 31.3% as FS failed to detect all positive cases, all cases with FN went delayed axillary dissection. False positive patients 5.8% didn't undergo dissection. |
| Soares 2007 | Retrospective | 36 | 30 | Paraffin section | Intraoperative evaluation of SLNs is a reliable method and should be performed by the cytologic smears instead of FS. |
| Somashekhar 2013 | Prospective | 164 | 368 | Paraffin section | IFS is a reliable method for detection of SLN metastasis that allows immediate axillary dissection in a one-stage procedure. However, FS may fail in micrometastasis detection. |
| Stovagraad 2012 | Retrospective | 336 | 335 | Paraffin section | Using immunohistochemistry (IHC) on FS increases detection of isolated tumor cell (ITCs) and micrometastasis intraoperatively. |
| Sun 2017 | Prospective | 79 | 342 | Paraffin section | FS was more accurate and specific but less sensitive than TIC. |
| Taffurelli 2012 | Prospective | 753 | 1.46 per case | Paraffin section | FS is highly effective in subgroup detection of SLN, so patients benefit from completion axillary lymph node dissection in one-stage surgical procedure. |
| Tan 2016 | Prospective | 81 | 80 | Paraffin section | Axillary lymph node dissection in case of SLN positive detected by FS may be limited in the future. |
| Tanis 2001 | Prospective | 262 | 406 | Paraffin section | FS allows immediate axillary lymph node dissection in most node-positive breast cancer patients |
| Tille 2009 | Retrospective | 160 | 361 | Paraffin section | FS is a reliable method for evaluation of SLN metastasis, allowing immediate dissection at the same operation. Our protocol determines a good sensitivity for micrometastasis detection. |
| Turner 1999 | Retrospective | 278 | 278 | Paraffin section | FS is reliable in detecting macrometastases SLN while there's high FN rate (50% in our experience) in micrometastases detection without using rapid IHC or step-section levels. |
| Upender 2009 | Prospective | 40 | 1-3 per case | Paraffin section | FS has slightly better sensitivity and accuracy than imprint smears. However, imprint cytology can be used if FS is unavailable. |
| Vanderveen 2008 | Prospective | 118 | 233 | Paraffin section | FS is a safe procedure to detect SLN metastasis and can spare the patients a second operation. FN occurs in patients with micrometastasis. |

SLN: sentinel lymph node; FS: frozen section; IFS: intraoperative frozen section; TIC: touch imprint cytology; TIHC: touch imprint cytology with immunohistochemical staining; FN: false negative; FNR: false negative rate; NAC: neoadjuvant chemotherapy; ITCs: isolated tumor cells; ALND: axillary lymph node dissection.

**Supplementary table 1, continues: Summary of the included studies.**

| Study ID | Design | Total No. of patients | Total No. of SLN | Comparator | Conclusion |
| --- | --- | --- | --- | --- | --- |
| Veronesi 1997 | Prospective | 163 | 160 | Permanent section | FS failed to detect 18 cases with micometastases detected by permanent section and they needed a second surgery. |
| Vohra 2015 | Prospective | 50 | 198 | Paraffin section | IFS is a reliable method in macrometastasis detection, allowing patients to have completion dissection in the same operation. |
| Vrande 2008 | Retrospective | 759 | 994 | Paraffin section | IFS is a useful method in SLN assessment and prediction of axillary lymph node status in breast cancer patients, allowing avoidance of a second operation. |
| Wada 2004 | Retrospective | 569 | 1098 | Permanent section | FS is a reliable procedure to detect SLN metastasis allowing patients with positive nodes to avoid second operation. However, FS fails in micrometastasis detection specially in small tumors. |
| Wang 2012 | Prospective | 552 | 1188 | Paraffin section | One-step nucleic acid amplification assay (OSNA) for SLN detection is more accurate and sensitive than FS and TIC, it can replace them in general practice. |
| Wang 2013 | Prospective | 479 | 1046 | Paraffin section | The GeneSearchTM Breast Lymph Node Assay is accurate and rapid for SLNs, and it can replace FS and TIC for application. |
| Weiser 2000 | Prospective | 890 | 231 | Paraffin section | FS fails to detect small metastases and its ability increases with tumor size |
| Wong 2014 | Retrospective | 2174 | 2202 | Paraffin section | FS failed to detect metastasis in more than half of the patients due to isolated tumor cells (ITCs) and micrometastasis. |
| Wong 2018 | Retrospective | 165 | 416 | Permanent section | FS sensitivity, specificity, and accuracy is similar in patients treated with NAC or without NAC. |
| Yang 2000 | Retrospective | 18 | 1.2 per case | Permanent section | FS is better than preoperative positron emission tomography (PET) in diagnosis of SLN detection. |
| Yoon 2019 | Retrospective | 4219 | 12422 | Paraffin section | IFS evaluation for SLN should not be routinely performed for all breast cancer patients. |
| Zurrida 2001 | Prospective | 192 | 192 | Paraffin section | IFS evaluation of SLN can successfully identify patients whom axillary dissection can be avoided. |

SLN: sentinel lymph node; FS: frozen section; IFS: intraoperative frozen section; TIC: touch imprint cytology; TIHC: touch imprint cytology with immunohistochemical staining; FN: false negative; FNR: false negative rate; NAC: neoadjuvant chemotherapy; ITCs: isolated tumor cells; ALND: axillary lymph node dissection.

**Supplementary table 2: The baseline characteristics of the included studies' population.^1-110^**

| Study ID | Age | Number of SLN | Radiology tumor size | | | | | Histologic Type | | | | | Estrogen receptor | | Progesterone  receptor | | HER-2 | | LVI | | Nuclear grade | | | Type of Metastasis | | |
| --- | --- | --- | --- | --- | --- | --- | --- | --- | --- | --- | --- | --- | --- | --- | --- | --- | --- | --- | --- | --- | --- | --- | --- | --- | --- | --- |
|  | Mean (SD) | Mean (range) | pTis | pT1 | pT2 | pT3 | pT4 | IDC | ILC | DCIS | Ducto-lobular | Other | +ve | -ve | +ve | -ve | +ve | -ve | +ve | -ve | G1 | G2 | G3 | MAC | MIC | ITCs |
| Abuoglu 2016 | 47.71 (10.42) | - | - | - | - | - | - | 48 | - | 23 | 1 | - | - | - | - | - | - | - | - | - | - | - | - | - | - | - |
| Agarwal 2005 | - | 1.38 (1-4) | 0 | 168 | 61 | 4 | 0 | - | - | - | - | - | - | - | - | - | - | - | 46 | 175 | 81 | 117 | 32 | - | - | - |
| Ahadi 2017 | 48.58 (11.62) | - | - | - | - | - | - | - | - | - | - | - | - | - | - | - | - | - | - | - | - | - | - | - | - | - |
| Aihara 2004 | 53 (9.5) | - | - | - | - | - | - | 90 | - | 5 | - | 12 | - | - | - | - | - | - | - | - | - | - | - | - | - | - |
| Ali 2008 | 60 (7.67) | 2.1 (1-6) | - | 37 | 43 | 14 | 0 | 76 | 18 | 0 | 0 | 0 | 78 | 16 | - | - | - | - | - | - | 9 | 50 | 35 | - | - | - |
| Al-Shibli 2005 | - | 2 (1-6) | - | - | - | - | - | 52 | 18 | 0 | 0 | 0 | - | - | - | - | - | - | - | - | - | - | - | 11 | 6 | 2 |
| Arlicot 2013 | - | 1.9 | 0 | 672 | 0 | 0 | 0 | 585 | 87 | 0 | 0 | 0 | - | - | - | - | - | - | - | - | - | - | - | 92 | 51 | 31 |
| Arora 2007 | 55 | 2 | - | - | - | - | - | 281 | 39 | - | - | - | 281 | - | 245 | - | - | - | - | - | - | - | - | - | 96 | - |
| Ballal 2017 | - | - | - | 37 | 55 | 3 | - | - | - | - | - | - | - | - | - | - | - | - | - | - | - | - | - | - | - | - |
| Ballehaninna 2013 | 57 (9.17) | 2.1 (1-7) | 209 | 51 | 7 | 0 | 0 | 0 | 0 | 267 | 0 | 0 | 208 | 35 | 165 | 77 | - | - | 5 | 262 | - | - | - | 3 | 6 | 4 |
| Barakat 2012 | 50.6 (10.83) | 3.8 | 46 | 173 | 199 | 15 | - | 358 | 41 | 46 | - | - | - | - | - | - | - | - | - | - | 39 | 214 | 140 | 116 | 45 | 0 |
| Bravo 2017 | 50.6 (9.67) | 2.17 (1-7) | - | 10 | 125 | 26 | 5 | 149 | 8 | - | - | 9 | 112 | 54 | 87 | 79 | 24 | 142 | 9 | 155 | 11 | 81 | 71 | 33 | 9 | 2 |
| Brogi 2005 | 57 (11.83) | 2.3 (1-7) | - | - | - | - | - | 12 | - | - | - | - | - | - | - | - | - | - | - | - | - | - | - | 25 | 25 | 0 |
| Celebioglua 2006 | 57 (8.67) | 1.8 | - | - | - | - | - | 90 | 9 | 0 | 2 | 1 | - | - | - | - | - | - | - | - | - | - | - | 27 | 20 | - |
| Chan 2011 | 53 (9.33) | 2.3 | - | 166 | 91 | 2 | - | 227 | 8 | - | - | 25 | - | - | - | - | - | - | - | - | - | - | - | 42 | 26 | 18 |
| Chao 2001 | 57.5 (10.67) | 2.6 | 9 | 140 | 43 | 3 | - | 152 | 19 | 15 | - | 12 | - | - | - | - | - | - | - | - | - | - | - | - | - | - |
| Choi 2006 | 48 (7.5) | 2.3 (1-5) | 3 | 56 | 20 | 0 | - | 70 | 2 | - | - | - | - | - | - | - | - | - | - | - | - | - | - | 12 | 4 | 4 |
| Cipolla 2010 | 58.6 (8.83) | 1.7 (1-6) | - | 264 | 98 | 6 | 0 | 290 | 37 | - | 8 | - | 328 | 26 | - | - | - | - | - | - | 78 | 197 | 86 | 66 | 2 | - |
| Cipolla 2020 | 58.7 (7.33) | 2.1 (1-11) | 0 | 1019 | 424 | 27 | 0 | 1196 | 117 | - | - | 157 | 1283 | 157 | 1181 | 255 | 529 | 902 | - | - | 255 | 838 | 307 | 239 | 12 | 2 |
| Cotarelo 2020 | 61.3 (11) | 2.1 (1-8) | - | 647 | 371 | 51 | 3 | 798 | 148 | - | - | 126 | - | - | - | - | - | - | - | - | 299 | 505 | 268 | 219 | 50 | 0 |
| Diest 1999 | 54 (10.5) | 1.37 | - | - | - | - | - | - | - | - | - | - | - | - | - | - | - | - | - | - | - | - | - | - | 4 | - |
| Elezoglu 2011 | 53 (11.7) | 2.5 (1-9) | - | - | - | - | - | 211 | - | - | - | - | - | - | - | - | - | - | - | - | - | - | - | - | - | - |
| Flett 1998 | 62 (13.25) | - | - | - | - | - | - | - | - | - | - | - | - | - | - | - | - | - | - | - | - | - | - | - | - | - |
| Frere-Belda 2012 | 58 (10.5) | - | - | 175 | 13 | 2 | 1 | 164 | 34 | 23 | 0 | 12 | 174 | 33 | 141 | 66 | 13 | 194 | 31 | 176 | 63 | 106 | 37 | 26 | 16 | 20 |
| Geertsema 2010 | 59.6 (11.32) | - | - | - | - | - | - | 744 | 108 | - | - | - | 759 | 119 | 584 | 293 | 75 | 667 | - | - | 368 | 337 | 164 | - | - | - |
| Gemignani 2000 | 57 | 1.9 (1-6) | 0 | 375 | 0 | 0 | 0 | - | - | - | - | - | - | - | - | - | - | - | - | - | - | - | - | - | 34 | - |
| Gipponi 2004 | 61.5 (6) | - | - | 252 | 78 | - | 4 | - | - | - | - | - | - | - | - | - | - | - | - | - | - | - | - | 82 | 23 | 0 |
| Grabau 2005 | 59 (9.8) | 2 (1-7) | - | - | - | - | - | 218 | 32 | - | - | 22 | - | - | - | - | - | - | - | - | 155 | 71 | 64 | 56 | 28 | 0 |
| Grabenstetter 2019 | 50 (9.67) | 3 (1-14) | - | - | - | - | - | 595 | 31 | - | 74 | - | 403 | 302 | 329 | 376 | 259 | 437 | 196 | 422 | 5 | 61 | 626 | 133 | 58 | 12 |
| Han 2013 | 46 (8.17) | - | 0 | 12 | 168 | 84 | 17 | - | - | - | - | - | 150 | 128 | 92 | 186 | 87 | 191 | - | - | - | - | - | - | - | - |
| Hashmi 2013 | - | - | - | - | - | - | - | 124 | 2 | - | - | - | - | - | - | - | - | - | 19 | 89 | 15 | 74 | 65 | 44 | 18 | 0 |
| Henry-Tillman 2002 | 58.2 (12.6) | - | - | 134 | - | - | - | 216 | 25 | - | - | - | - | - | - | - | - | - | - | - | - | - | - | - | - | - |
| Hill 1998 | 56 (11) | 2.1 (1-8) | - | - | - | - | - | - | - | - | - | - | - | - | - | - | - | - | - | - | - | - | - | - | - | - |

Age is presented as mean (SD), SLN as mean (range) and all other data as frequency.

MAC: macro-metastasis; MIC: micro-metastasis; ITCs: isolated tumor cells; SLN: sentinel lymph nodes; IDC: infiltrating ductal carcinoma; ILC: infiltrating lobular carcinoma; DCIS: ductal carcinoma in situ; HER-2: human epidermal growth factor receptor 2; LVI: lympho-vascular invasion; +ve: positive; -ve: negative; G: grade.

**Supplementary table 2, continues: The baseline characteristics of the included studies' population.**

| Study ID | Age | Number of SLN | Radiology tumor size | | | | | Histologic Type | | | | | Estrogen receptor | | Progesterone  receptor | | HER-2 | | LVI | | Nuclear grade | | | Type of Metastasis | | |
| --- | --- | --- | --- | --- | --- | --- | --- | --- | --- | --- | --- | --- | --- | --- | --- | --- | --- | --- | --- | --- | --- | --- | --- | --- | --- | --- |
|  | Mean (SD) | Mean (range) | pTis | pT1 | pT2 | pT3 | pT4 | IDC | ILC | DCIS | Ducto-lobular | Other | +ve | -ve | +ve | -ve | +ve | -ve | +ve | -ve | G1 | G2 | G3 | MAC | MIC | ITCs |
| Hino 2008 | 48 (11.5) | - | 0 | 0 | 33 | 22 | 0 | 54 | 1 | 0 | 0 | 0 | 18 | 37 | - | - | - | - | - | - | - | - | - | 18 | 0 | 0 |
| Holck 2004 | - | - | - | - | - | - | - | - | - | - | - | - | - | - | - | - | - | - | - | - | - | - | - | - | - | - |
| Horvath 2009 | 57 (10.5) | - | 0 | 163 | 92 | 7 | 1 | 133 | 131 | 0 | 0 | 0 | 217 | 31 | 189 | 60 | 48 | 193 | - | - | 72 | 57 | 17 | 64 | 28 | - |
| Houpu 2019 | 54.7 (0.2) | - | 259 | 1701 | 516 | 18 | - | 1628 | 582 | - | - | - | 1890 | 647 | 1638 | 899 | 444 | 1878 | 426 | 1965 | 237 | 1077 | 429 | 541 | 54 | 21 |
| Hung 2005 | 52.5 | 2.5 (1-6) | - | - | - | - | - | - | - | - | - | - | - | - | - | - | - | - | - | - | - | - | - | - | - | - |
| Imoto 2000 | - | - | - | 12 | 46 | 1 | - | - | - | - | - | - | - | - | - | - | - | - | - | - | - | - | - | - | 2 | - |
| Jaka 2010 | 54.81 (10.67) | - | - | - | - | - | - | 77 | 7 | 3 | 1 | 27 | - | - | - | - | 56 | 34 | 53 | 31 | 1 | 14 | 68 | - | - | - |
| Jamal 2011 | 56.8 (9.67) | 2.11 (1-6) | - | - | - | - | - | 133 | 12 | - | 21 | 9 | 144 | 32 | 110 | 66 | 16 | 160 | 37 | 139 | 23 | 112 | 16 | 22 | 26 | - |
| Jara-Lazaro 2014 | - | 1.7 (1-4) | - | - | - | - | - | 39 | 2 | 5 | 1 | 7 | - | - | - | - | - | - | - | - | - | - | - | - | - | - |
| Jylling 2008 | - | - | - | - | - | - | - | - | - | - | - | - | - | - | - | - | - | - | - | - | - | - | - | 2 | 44 | 24 |
| Kelley 1999 | - | - | - | - | - | - | - | - | - | - | - | - | - | - | - | - | - | - | - | - | - | - | - | - | - | - |
| Khalifa 2004 | 65 (8) | - | - | - | - | - | - | - | - | - | - | - | - | - | - | - | - | - | - | - | - | - | - | - | - | - |
| krishnamurthy 2009 | - | - | - | - | - | - | - | 85 | 8 | 0 | 5 | 2 | - | - | - | - | - | - | - | - | 18 | 41 | 41 | 12 | 3 | - |
| Krogerus 2004 | 55.5 (8.8) | - | 12 | 143 | 47 | 0 | 2 | 99 | 64 | 12 | 0 | 28 | - | - | - | - | - | - | - | - | 77 | 87 | 39 | - | 21 | 4 |
| Lai 2018 | 55 (9.5) | - | 8 | 34 | 32 | 3 | 0 | - | 1 | 8 | - | - | - | - | - | - | - | - | - | - | 25 | 30 | 13 | 8 | 6 | 1 |
| Langer 2009 | 58.9 (10.33) | - | 0 | 458 | 190 | 0 | 0 | 561 | 72 | - | - | 15 | 558 | 90 | 500 | 148 | - | - | - | - | 123 | 385 | 140 | 145 | 68 | 19 |
| Lauridsen 2004 | 56 (11) | 2 (1-7) | - | - | - | - | - | - | - | - |  | - | - | - | - | - | - | - | - | - | - | - | - | - | - | - |
| Lee 2006 | 47.7 (11.5) | 1.44 (1-4) | 0 | 37 | 23 | 0 | 0 | 52 | 2 | 2 | 0 | 6 | - | - | - | - | - | - | - | - | - | - | - | - | - | - |
| Leidenius 2003 | 56 (9.17) | 2 (1-14) | 11 | 272 | 89 | 3 | - | 194 | 102 | 11 | 0 | 68 | - | - | - | - | - | - | - | - | 134 | 157 | 70 | 95 | 43 | - |
| Leung 2007 | - | 3.9 (1-10) | 38 | 136 | 120 | 6 | 0 | - | - | - | - | - | - | - | - | - | - | - | 126 | 136 | - | - | - | - | - | - |
| Liang 2003 | 59 | 2.1 (0-4) | - | - | - | - | - | - | - | - | - | - | - | - | - | - | - | - | - | - | - | - | - | - | - | - |
| Lim 2013 | 60 (11) | 1.57 (0-7) | - | 361 | 184 | 12 | 3 | 523 | 71 | 42 | 45 | - | - | - | - | - | - | - | - | - | 95 | 364 | 204 | 156 | 8 | - |
| Liu 2000 | 59 (10.75) | 1.5 (1-4) | - | - | - | - | - | 25 | 5 | 0 | 4 | 4 | - | - | - | - | - | - | - | - | - | - | - | - | - | - |
| Liu 2011 | 55.7 (10..83) | - | 0 | 265 | 57 | 4 | 0 | 278 | 29 | - | - | 19 | - | - | - | - | - | - | - | - | - | - | - | 61 | 38 | - |
| Lombardi 2018 | 57 (10.67) | - | - | - | - | - | - | - | - | - | - | - | - | - | - | - | - | - | - | - | - | - | - | - | - | - |
| Lu 2013 | 54 (10.83) | 2 (1-8) | - | - | - | - | - | 408 | 35 | 60 | - | 39 | - | - | - | - | - | - | - | - | 115 | 222 | 201 | - | - | - |
| Lumachi 2011 | 56 (7) | - | - | - | - | - | - | - | - | - | - | - | 83 | 29 | - | - | - | - | - | - | 26 | 64 | 19 | - | - | - |
| Lumachi 2012 | 52 (6.2) | 2 (1-5) | - | - | - | - | - | 98 | 11 | - | - | 17 | - | - | - | - | - | - | - | - | - | - | - | 51 | - | - |
| Mclaughlin 2008 | 57 (10.5) | - | - | - | - | - | - | 770 | 88 | - | - | - | 743 | 188 | - | - | - | - | 227 | 704 | - | - | - | - | - | - |
| Memar 2010 | 47 (11.88) | 1 | - | - | - | - | - | 72 | 24 | 0 | 0 | 1 | - | - | - | - | - | - | - | - | - | - | - | - | - | - |
| Menes 2003 | 55 (9.83) | - | - | - | - | - | - | 69 | 11 | 5 | 25 | 8 | - | - | - | - | - | - | - | - | - | - | - | 23 | 10 | - |
| Mitchell 2005 | 56 (7.67) | 2.4 (1-12) | - | - | - | - | - | - | - | - | - | - | - | - | - | - | - | - | - | - | - | - | - | - | - | - |
| Moatasim 2013 | 49.81 (11.1) | 2 (1-6) | - | - | - | - | - | 75 | 2 | 6 | 1 | 9 | - | - | - | - | - | - | - | - | - | - | - | 11 | 25 | 0 |
| Morgan 1999 | 65 (11.75) | 1.1 (1-2) | 0 | 34 | 10 | 0 | 0 | 37 | 6 | 0 | 0 | 1 | - | - | - | - | - | - | - | - | - | - | - | - | - | - |

Age is presented as mean (SD), SLN as mean (range) and all other data as frequency.

MAC: macro-metastasis; MIC: micro-metastasis; ITCs: isolated tumor cells; SLN: sentinel lymph nodes; IDC: infiltrating ductal carcinoma; ILC: infiltrating lobular carcinoma; DCIS: ductal carcinoma in situ; HER-2: human epidermal growth factor receptor 2; LVI: lympho-vascular invasion; +ve: positive; -ve: negative; G: grade.

**Supplementary table 2, continues: The baseline characteristics of the included studies' population.**

| Study ID | Age | Number of SLN | Radiology tumor size | | | | | Histologic Type | | | | | Estrogen receptor | | Progesterone  receptor | | HER-2 | | LVI | | Nuclear grade | | | Type of Metastasis | | |
| --- | --- | --- | --- | --- | --- | --- | --- | --- | --- | --- | --- | --- | --- | --- | --- | --- | --- | --- | --- | --- | --- | --- | --- | --- | --- | --- |
|  | Mean (SD) | Mean (range) | pTis | pT1 | pT2 | pT3 | pT4 | IDC | ILC | DCIS | Ducto-lobular | Other | +ve | -ve | +ve | -ve | +ve | -ve | +ve | -ve | G1 | G2 | G3 | MAC | MIC | ITCs |
| Mori 2006 | - | 1.7 | - | - | - | - | - | - | - | - | - | - | - | - | - | - | - | - | - | - | - | - | - | - | - | - |
| Motomura 2000 | 51 (7.83) | 1.6 (1-6) | - | - | - | - | - | - | - | - | - | - | - | - | - | - | - | - | - | - | - | - | - | - | - | - |
| Nagashima 2003 | 56 (8.33) | 2.4 | - | - | - | - | - | 101 | - | - | - | - | - | - | - | - | - | - | - | - | - | - | - | - | 8 | - |
| Nährig 2003 | 48 (11.75) | 1.1 | 0 | 30 | 20 | 0 | 0 | 45 | 4 | 0 | 0 | 1 | - | - | - | - | - | - | - | - | - | - | - | 6 | 3 | 6 |
| Nofech-Mozes 2009 | 58 (10.67) | 2.4 (1-12) | - | - | - | - | - | 581 | 63 | - | - | - | - | - | - | - | - | - | - | - | - | - | - | 675 | 21 | 10 |
| Noguchi 2000 | 53 (12) | - | - | 77 | 63 | - | - | 118 | - | - | - | - | - | - | - | - | - | - | - | - | - | - | - | - | - | - |
| Nowikiewicz 2015 | 56.6 (9.5) | - | - | 180 | 101 | 4 | - | 252 | 28 | - | - | - | 246 | 39 | - | - | 37 | 246 | - | - | 25 | 187 | 65 | 222 | 60 | 3 |
| Perez 2005 | 59.2 (8.5) | 1.65 (1-5) | - | - | - | - | - | 77 | 3 | 0 | 0 | 7 | - | - | - | - | - | - | - | - | - | - | - | 23 | 2 | 0 |
| Petropoulou 2017 | 61.3 (12.75) | 1.33 | - | - | - | - | - | - | - | - | - | - | 53 | 7 | 43 | 17 | - | - | - | - | - | - | - | 7 | 2 | 1 |
| Poling 2014 | - | - | - | - | - | - | - | - | - | - | - | - | - | - | - | - | - | - | - | - | - | - | - | 29 | 40 | 26 |
| Qiao 2016 | - | - | - | - | - | - | - | - | - | - | - | - | - | - | - | - | - | - | - | - | - | - | - | 9 | 33 | 11 |
| Rahusen 2000 | 54 (8.5) | 1.6 (1-4) | - | - | - | - | - | - | - | 8 | - | - | - | - | - | - | - | - | - | - | - | - | - | 19 | 18 | 0 |
| Reitsamer 2004 | - | - | 0 | 219 | 108 | 1 | 0 | 294 | 34 | 0 | 0 | 0 | 289 | 39 | 260 | 68 | - | - | - | - | 30 | 215 | 83 | - | - | - |
| Rubio 2010 | 50.5 (9.8) | - | - | - | - | - | - | 385 | 50 | - | - | - | - | - | - | - | - | - | - | - | - | - | - | 2 | 15 | 2 |
| Russo 2017 | 54 (11) | - | - | 136 | 134 | 9 | 0 | 224 | 18 | - | 12 | - | - | - | - | - | - | - | 114 | 167 | 52 | 185 | 44 | 61 | 15 | 0 |
| Safai 2012 | 45.29 (10.6) | 2 | - | - | - | - | - | - | - | - | - | - | - | - | - | - | - | - | - | - | - | - | - | - | - | - |
| Schrnek 2004 | 51.6 (10.5) | - | - | 35 | 44 | - | - | 63 | 15 | 1 | - | - | 62 | 18 | 48 | 32 | 61 | 19 | 6 | 74 | - | - | - | 23 | 12 | 0 |
| Schwartz 2008 | 59 (8.17) | - | - | - | - | - | - | 199 | 15 | 10 | - | - | - | - | - | - | - | - | - | - | 30 | 112 | 75 | 1 | 9 | 1 |
| Shimazu 2008 | 53.4 (10.9) | - | - | 165 | 181 | 15 | 2 | 333 | 19 | - | - | - | - | - | - | - | - | - | - | - | - | - | - | 78 | 38 | - |
| Shojaee 2020 | 49 (8.17) | - | - | - | - | - | - | 87 | - | - | - | - | - | - | - | - | 31 | - | - | - | - | - | - | - | - | - |
| Soares 2007 | 58.4 (14) | - | - | 16 | 17 | 3 | - | 17 | 4 | - | - | - | - | - | - | - | - | - | - | - | - | - | - | 15 | 1 | 2 |
| Somasherhs 2013 | 54 (9.17) | 2 (1-5) | - | 62 | 96 | - | - | - | - | - | - | - | - | - | - | - | - | - | 70 | 88 | 12 | 58 | 88 | - | - | - |
| Stovagraad 2012 | - | - | - | - | - | - | - | - | - | - | - | - | - | - | - | - | - | - | - | - | - | - | - | 80 | 25 | 13 |
| Sun 2017 | 50 (8.33) | 4.3 | - | - | - | - | - | 74 | 2 | - | - | - | 61 | - | 51 | - | - | - | 0 | 79 | - | - | - | - | - | - |
| Taffurelli 2012 | - | 1.4 | 56 | 622 | 74 | 1 | 0 | - | - | - | - | - | - | - | - | - | - | - | - | - | - | - | - | 129 | 27 | 2 |
| Tan 2016 | 63 (7.17) | - | 7 | 58 | 14 | 1 | 0 | 57 | 10 | 4 | - | - | - | - | - | - | - | - | - | - | 31 | 28 | 15 | - | - | - |
| Tanis 2001 | - | 1.7 (1-6) | 0 | 178 | 86 | 1 | 0 | 214 | 34 | - | - | - | - | - | - | - | - | - | - | - | - | - | - | - | - | - |
| Tille 2009 | 55 (11.5) | 2.25 (1-9) | - | 116 | 33 | - | - | 119 | 15 | - | - | - | 130 | 19 | 113 | 36 | - | - | 18 | 131 | 46 | 84 | 19 | 42 | 10 | 11 |
| Turner 1999 | 56.5 (9.3) | 1.4 | 0 | 207 | 66 | 5 | 0 | 227 | 27 | 8 | 0 | 16 | 225 | 53 | 182 | 96 | - | - | - | - | - | - | - | 47 | 64 | 0 |
| Upender 2009 | 49.8 (11.19) | - | - | 24 | 16 | 0 | 0 | 37 | 2 | 0 | 0 | 1 | - | - | - | - | - | - | 13 | 27 | 9 | 21 | 7 | 17 | 6 | 1 |
| Vanderveen 2008 | 59 (10.83) | - | - | - | - | - | - | 89 | 4 | 4 | 14 | 7 | 96 | 22 | 75 | 43 | 19 | 99 | 29 | 81 | 36 | 54 | 20 | - | - | - |
| Veronesi 1997 | 51 (8.67) | - | - | - | - | - | - | - | - | - | - | - | 120 | 43 | - | - | - | - | - | - | - | - | - | - | 18 | - |
| Vohra 2015 | 55 (11.75) | - | 0 | 2 | 44 | 4 | 0 | 46 | 3 | 0 | 0 | 1 | 42 | 8 | 40 | 10 | - | - | - | - | 2 | 46 | 2 | - | - | - |
| Vrande 2008 | 59 (10.17) | 1.6 | - | 447 | 162 | 3 | - | 453 | 98 | 299 | 3 | - | - | - | - | - | - | - | - | - | - | - | - | 81 | 95 | 16 |
| Wada 2004 | 54 (10.5) | 1.9 | 24 | 246 | 299 | 0 | 0 | 448 | 25 | - | - | 72 | - | - | - | - | - | - | - | - | - | - | - | - | 19 | - |

Age is presented as mean (SD), SLN as mean (range) and all other data as frequency.

MAC: macro-metastasis; MIC: micro-metastasis; ITCs: isolated tumor cells; SLN: sentinel lymph nodes; IDC: infiltrating ductal carcinoma; ILC: infiltrating lobular carcinoma; DCIS: ductal carcinoma in situ; HER-2: human epidermal growth factor receptor 2; LVI: lympho-vascular invasion; +ve: positive; -ve: negative; G: grade.

**Supplementary table 2 continues: The baseline characteristics of the included studies' population.**

| Study ID | Age | Number of SLN | Radiology tumor size | | | | | Histologic Type | | | | | Estrogen receptor | | Progesterone  receptor | | HER-2 | | LVI | | Nuclear grade | | | Type of Metastasis | | |
| --- | --- | --- | --- | --- | --- | --- | --- | --- | --- | --- | --- | --- | --- | --- | --- | --- | --- | --- | --- | --- | --- | --- | --- | --- | --- | --- |
|  | Mean (SD) | Mean (range) | pTis | pT1 | pT2 | pT3 | pT4 | IDC | ILC | DCIS | Ducto-lobular | Other | +ve | -ve | +ve | -ve | +ve | -ve | +ve | -ve | G1 | G2 | G3 | MAC | MIC | ITCs |
| Wada 2004 | 54 (10.5) | 1.9 | 24 | 246 | 299 | 0 | 0 | 448 | 25 | - | - | 72 | - | - | - | - | - | - | - | - | - | - | - | - | 19 | - |
| Wang 2012 | 50 (9.83) | - | 74 | 250 | 173 | 10 | - | 383 | 23 | 70 | - | - | 382 | 138 | - | - | 179 | 335 | - | - | - | - | - | 31 | 107 | - |
| Wang 2013 | 48 (10.67) | 2.18 | 48 | 147 | 254 | 30 | - | 340 | 30 | 48 | - | - | 319 | 160 | - | - | 163 | 316 | - | - | - | - | - | 29 | 99 | 0 |
| Weiser 2000 | 53 (10.67) | - | 0 | 771 | 119 | 0 | 0 | 202 | 24 | - | - | - | - | - | - | - | - | - | - | - | - | - | - | 109 | 115 | - |
| Wong 2014 | 55 (10.83) | - | - | 992 | 973 | 104 | - | 1801 | 98 | - | 22 | - | 1495 | 456 | 1384 | 566 | 427 | 1481 | 442 | 1759 | 305 | 818 | 898 | 549 | 82 | 29 |
| Wong 2018 | - | - | - | - | - | - | - | 140 | 12 | - | - | - | 117 | 47 | - | - | 33 | 128 | - | - | 15 | 58 | 68 | 36 | 13 | 11 |
| Yang 2000 | 45 (9) | 1.3 (1-3) | 2 | 7 | 7 | 2 | 0 | 15 | 0 | 2 | 0 | 1 | - | - | - | - | - | - | - | - | 6 | 8 | 4 | - | - | - |
| Yoon 2019 | 51.8 (10.78) | 2.8 | 0 | 3163 | 1020 | 36 | 0 | 3559 | 209 | - | - | - | 3203 | 1007 | - | - | 970 | 3249 | 329 | 3890 | 1096 | 2169 | 954 | - | 234 | - |
| Zurrida 2001 | - | - | - | 143 | - | - | - | - | - | - | - | - | - | - | - | - | - | - | - | - | - | - | - | - | 35 | - |

Age is presented as mean (SD), SLN as mean (range) and all other data as frequency.

MAC: macro-metastasis; MIC: micro-metastasis; ITCs: isolated tumor cells; SLN: sentinel lymph nodes; IDC: infiltrating ductal carcinoma; ILC: infiltrating lobular carcinoma; DCIS: ductal carcinoma in situ; HER-2: human epidermal growth factor receptor 2; LVI: lympho-vascular invasion; +ve: positive; -ve: negative; G: grade.

**References**

1. Veronesi U, Paganelli G, Galimberti V, et al. Sentinel-node biopsy to avoid axillary dissection in breast cancer with clinically negative lymph-nodes. *Lancet (London, England)*. Jun 28 1997;349(9069):1864-7. doi:10.1016/s0140-6736(97)01004-0

2. Flett MM, Going JJ, Stanton PD, Cooke TG. Sentinel node localization in patients with breast cancer. *The British journal of surgery*. Jul 1998;85(7):991-3. doi:10.1046/j.1365-2168.1998.00746.x

3. Hill AD, Tran KN, Akhurst T, et al. Lessons learned from 500 cases of lymphatic mapping for breast cancer. *Annals of surgery*. Apr 1999;229(4):528-35. doi:10.1097/00000658-199904000-00012

4. Kelley SW, Komorowski RA, Dayer AM. Axillary sentinel lymph node examination in breast carcinoma. *Archives of pathology & laboratory medicine*. Jun 1999;123(6):533-5. doi:10.1043/0003-9985(1999)123<0533:aslnei>2.0.co;2

5. Morgan A, Howisey RL, Aldape HC, et al. Initial experience in a community hospital with sentinel lymph node mapping and biopsy for evaluation of axillary lymph node status in palpable invasive breast cancer. *Journal of surgical oncology*. Sep 1999;72(1):24-30; discussion 30-1. doi:10.1002/(sici)1096-9098(199909)72:1<24::aid-jso6>3.0.co;2-x

6. Turner RR, Hansen NM, Stern SL, Giuliano AE. Intraoperative examination of the sentinel lymph node for breast carcinoma staging. *American journal of clinical pathology*. Nov 1999;112(5):627-34. doi:10.1093/ajcp/112.5.627

7. Van Diest PJ, Torrenga H, Borgstein PJ, et al. Reliability of intraoperative frozen section and imprint cytological investigation of sentinel lymph nodes in breast cancer. *Histopathology*. Jul 1999;35(1):14-8. doi:10.1046/j.1365-2559.1999.00667.x

8. Gemignani ML, Cody HS, 3rd, Fey JV, Tran KN, Venkatraman E, Borgen PI. Impact of sentinel lymph node mapping on relative charges in patients with early-stage breast cancer. *Annals of surgical oncology*. Sep 2000;7(8):575-80. doi:10.1007/bf02725336

9. Imoto S, Fukukita H, Murakami K, Ikeda H, Moriyama N. Pilot study on sentinel node biopsy in breast cancer. *Journal of surgical oncology*. Mar 2000;73(3):130-3. doi:10.1002/(sici)1096-9098(200003)73:3<130::aid-jso2>3.0.co;2-x

10. Liu LH, Siziopikou KP, Gabram S, McClatchey KD. Evaluation of axillary sentinel lymph node biopsy by immunohistochemistry and multilevel sectioning in patients with breast carcinoma. *Archives of pathology & laboratory medicine*. Nov 2000;124(11):1670-3. doi:10.1043/0003-9985(2000)124<1670:eoasln>2.0.co;2

11. Motomura K, Inaji H, Komoike Y, et al. Intraoperative sentinel lymph node examination by imprint cytology and frozen sectioning during breast surgery. *The British journal of surgery*. May 2000;87(5):597-601. doi:10.1046/j.1365-2168.2000.01423.x

12. Noguchi M, Tsugawa K, Miwa K, et al. Sentinel lymph node biopsy in breast cancer using blue dye with or without isotope localization. *Breast cancer (Tokyo, Japan)*. 2000;7(4):287-96. doi:10.1007/bf02966392

13. Rahusen FD, Pijpers R, Van Diest PJ, Bleichrodt RP, Torrenga H, Meijer S. The implementation of the sentinel node biopsy as a routine procedure for patients with breast cancer. *Surgery*. Jul 2000;128(1):6-12. doi:10.1067/msy.2000.107229

14. Weiser MR, Montgomery LL, Susnik B, Tan LK, Borgen PI, Cody HS. Is routine intraoperative frozen-section examination of sentinel lymph nodes in breast cancer worthwhile? *Annals of surgical oncology*. Oct 2000;7(9):651-5. doi:10.1007/s10434-000-0651-3

15. Chao C, Wong SL, Ackermann D, et al. Utility of intraoperative frozen section analysis of sentinel lymph nodes in breast cancer. *American journal of surgery*. Dec 2001;182(6):609-15. doi:10.1016/s0002-9610(01)00794-2

16. Tanis PJ, Boom RP, Koops HS, et al. Frozen section investigation of the sentinel node in malignant melanoma and breast cancer. *Annals of surgical oncology*. Apr 2001;8(3):222-6. doi:10.1007/s10434-001-0222-2

17. Yang JH, Nam SJ, Lee TS, Lee HK, Jung SH, Kim BT. Comparison of intraoperative frozen section analysis of sentinel node with preoperative positron emission tomography in the diagnosis of axillary lymph node status in breast cancer patients. *Japanese journal of clinical oncology*. Jan 2001;31(1):1-6. doi:10.1093/jjco/hye007

18. Zurrida S, Mazzarol G, Galimberti V, et al. The problem of the accuracy of intraoperative examination of axillary sentinel nodes in breast cancer. *Annals of surgical oncology*. Dec 2001;8(10):817-20. doi:10.1007/s10434-001-0817-7

19. Henry-Tillman RS, Korourian S, Rubio IT, et al. Intraoperative touch preparation for sentinel lymph node biopsy: a 4-year experience. *Annals of surgical oncology*. May 2002;9(4):333-9. doi:10.1007/bf02573867

20. Leidenius MH, Krogerus LA, Toivonen TS, Von Smitten KJ. The feasibility of intraoperative diagnosis of sentinel lymph node metastases in breast cancer. *Journal of surgical oncology*. Oct 2003;84(2):68-73. doi:10.1002/jso.10296

21. Liang R, Craik J, Juhasz ES, Harman CR. Imprint cytology versus frozen section: intraoperative analysis of sentinel lymph nodes in breast cancer. *ANZ journal of surgery*. Aug 2003;73(8):597-9. doi:10.1046/j.1445-2197.2003.02728.x

22. Menes TS, Tartter PI, Mizrachi H, Smith SR, Estabrook A. Touch preparation or frozen section for intraoperative detection of sentinel lymph node metastases from breast cancer. *Annals of surgical oncology*. Dec 2003;10(10):1166-70. doi:10.1245/aso.2003.04.023

23. Nagashima T, Suzuki M, Yagata H, et al. Intraoperative cytologic diagnosis of sentinel node metastases in breast cancer. *Acta cytologica*. Nov-Dec 2003;47(6):1028-32. doi:10.1159/000326640

24. Nahrig JM, Richter T, Kuhn W, et al. Intraoperative examination of sentinel lymph nodes by ultrarapid immunohistochemistry. *The breast journal*. Jul-Aug 2003;9(4):277-81. doi:10.1046/j.1524-4741.2003.09405.x

25. Aihara T, Munakata S, Morino H, Takatsuka Y. Comparison of frozen section and touch imprint cytology for evaluation of sentinel lymph node metastasis in breast cancer. *Annals of surgical oncology*. Aug 2004;11(8):747-50. doi:10.1245/aso.2004.01.014

26. Gipponi M, Bassetti C, Canavese G, et al. Sentinel lymph node as a new marker for therapeutic planning in breast cancer patients. *Journal of surgical oncology*. Mar 2004;85(3):102-11. doi:10.1002/jso.20022

27. Holck S, Galatius H, Engel U, Wagner F, Hoffmann J. False-negative frozen section of sentinel lymph node biopsy for breast cancer. *Breast (Edinburgh, Scotland)*. Feb 2004;13(1):42-8. doi:10.1016/s0960-9776(03)00124-3

28. Khalifa K, Pereira B, Thomas VA, Mokbel K. The accuracy of intraoperative frozen section analysis of the sentinel lymph nodes during breast cancer surgery. *International journal of fertility and women's medicine*. Sep-Oct 2004;49(5):208-11.

29. Krogerus LA, Leidenius MH, Toivonen TS, von Smitten KJ. Towards reasonable workload in diagnosis of sentinel lymph nodes: comparison of two frozen section methods. *Histopathology*. Jan 2004;44(1):29-34. doi:10.1111/j.1365-2559.2004.01746.x

30. Lauridsen MC, Garne JP, Sorensen FB, Melsen F, Lernevall A, Christiansen P. Sentinel lymph node biopsy in breast cancer--experience with the combined use of dye and radioactive tracer at Aarhus University Hospital. *Acta oncologica (Stockholm, Sweden)*. 2004;43(1):20-6. doi:10.1080/02841860310017757

31. Reitsamer R, Peintinger F, Prokop E, Rettenbacher L, Menzel C. 200 Sentinel lymph node biopsies without axillary lymph node dissection -- no axillary recurrences after a 3-year follow-up. *British journal of cancer*. Apr 19 2004;90(8):1551-4. doi:10.1038/sj.bjc.6601765

32. Wada N, Imoto S, Hasebe T, Ochiai A, Ebihara S, Moriyama N. Evaluation of intraoperative frozen section diagnosis of sentinel lymph nodes in breast cancer. *Japanese journal of clinical oncology*. Mar 2004;34(3):113-7. doi:10.1093/jjco/hyh023

33. Agarwal T, Kakkos SK, Cunningham DA, et al. Sentinel node biopsy can replace four-node-sampling in staging early breast cancer. *European journal of surgical oncology : the journal of the European Society of Surgical Oncology and the British Association of Surgical Oncology*. Mar 2005;31(2):122-7. doi:10.1016/j.ejso.2004.09.010

34. Al-Shibli KI, Mohammed HA, Mikalsen KS. Sentinel lymph nodes and breast carcinoma: analysis of 70 cases by frozen section. *Annals of Saudi medicine*. Mar-Apr 2005;25(2):111-4. doi:10.5144/0256-4947.2005.111

35. Brogi E, Torres-Matundan E, Tan LK, Cody HS, 3rd. The results of frozen section, touch preparation, and cytological smear are comparable for intraoperative examination of sentinel lymph nodes: a study in 133 breast cancer patients. *Annals of surgical oncology*. Feb 2005;12(2):173-80. doi:10.1245/aso.2005.03.067

36. Grabau DA, Rank F, Friis E. Intraoperative frozen section examination of axillary sentinel lymph nodes in breast cancer. *APMIS : acta pathologica, microbiologica, et immunologica Scandinavica*. Jan 2005;113(1):7-12. doi:10.1111/j.1600-0463.2005.apm1130102.x

37. Hung WK, Chan CM, Ying M, Chong SF, Mak KL, Yip AW. Randomized clinical trial comparing blue dye with combined dye and isotope for sentinel lymph node biopsy in breast cancer. *The British journal of surgery*. Dec 2005;92(12):1494-7. doi:10.1002/bjs.5211

38. Mitchell ML. Frozen section diagnosis for axillary sentinel lymph nodes: the first six years. *Modern pathology : an official journal of the United States and Canadian Academy of Pathology, Inc*. Jan 2005;18(1):58-61. doi:10.1038/modpathol.3800279

39. Perez N, Vidal-Sicart S, Zanon G, et al. A practical approach to intraoperative evaluation of sentinel lymph node biopsy in breast carcinoma and review of the current methods. *Annals of surgical oncology*. Apr 2005;12(4):313-21. doi:10.1245/aso.2005.07.003

40. Schrenk P, Woelfl S, Bogner S, Moser F, Wayand W. The use of sentinel node biopsy in breast cancer patients undergoing skin sparing mastectomy and immediate autologous reconstruction. *Plastic and reconstructive surgery*. Oct 2005;116(5):1278-86. doi:10.1097/01.prs.0000181515.11529.9a

41. Celebioglu F, Sylvan M, Perbeck L, Bergkvist L, Frisell J. Intraoperative sentinel lymph node examination by frozen section, immunohistochemistry and imprint cytology during breast surgery--a prospective study. *European journal of cancer (Oxford, England : 1990)*. Mar 2006;42(5):617-20. doi:10.1016/j.ejca.2005.12.003

42. Choi YJ, Yun HR, Yoo KE, et al. Intraoperative examination of sentinel lymph nodes by ultrarapid immunohistochemistry in breast cancer. *Japanese journal of clinical oncology*. Aug 2006;36(8):489-93. doi:10.1093/jjco/hyl045

43. Lee IK, Lee HD, Jeong J, et al. Intraoperative examination of sentinel lymph nodes by immunohistochemical staining in patients with breast cancer. *European journal of surgical oncology : the journal of the European Society of Surgical Oncology and the British Association of Surgical Oncology*. May 2006;32(4):405-9. doi:10.1016/j.ejso.2006.01.009

44. Mori M, Tada K, Ikenaga M, et al. Frozen section is superior to imprint cytology for the intra-operative assessment of sentinel lymph node metastasis in stage I breast cancer patients. *World journal of surgical oncology*. May 17 2006;4:26. doi:10.1186/1477-7819-4-26

45. Leung KM, Chan KW, Yeoh GP, Chan JK, Cheung PS. Clinical relevance of intra-operative sentinel lymph node examination in breast cancer management. *Hong Kong medical journal = Xianggang yi xue za zhi*. Feb 2007;13(1):8-11.

46. Soares CT, Frederigue-Junior U, Luca LA. Anatomopathological analysis of sentinel and nonsentinel lymph nodes in breast cancer: hematoxylin-eosin versus immunohistochemistry. *International journal of surgical pathology*. Oct 2007;15(4):358-68. doi:10.1177/1066896907302124

47. Ali R, Hanly AM, Naughton P, et al. Intraoperative frozen section assessment of sentinel lymph nodes in the operative management of women with symptomatic breast cancer. *World journal of surgical oncology*. Jun 26 2008;6:69. doi:10.1186/1477-7819-6-69

48. Arora N, Martins D, Huston TL, et al. Sentinel node positivity rates with and without frozen section for breast cancer. *Annals of surgical oncology*. Jan 2008;15(1):256-61. doi:10.1245/s10434-007-9600-8

49. Hino M, Sano M, Sato N, Homma K. Sentinel lymph node biopsy after neoadjuvant chemotherapy in a patient with operable breast cancer. *Surgery today*. 2008;38(7):585-91. doi:10.1007/s00595-007-3686-2

50. Jylling AM, Lindebjerg J, Nielsen L, Jensen J. Immunohistochemistry on frozen section of sentinel lymph nodes in breast cancer with improved morphology and blocking of endogenous peroxidase. *Applied immunohistochemistry & molecular morphology : AIMM*. Oct 2008;16(5):482-4. doi:10.1097/PAI.0b013e3181600023

51. McLaughlin SA, Ochoa-Frongia LM, Patil SM, Cody HS, 3rd, Sclafani LM. Influence of frozen-section analysis of sentinel lymph node and lumpectomy margin status on reoperation rates in patients undergoing breast-conservation therapy. *Journal of the American College of Surgeons*. Jan 2008;206(1):76-82. doi:10.1016/j.jamcollsurg.2007.07.021

52. Schwartz GF, Krill LS, Palazzo JP, Dasgupta A. Value of intraoperative examination of axillary sentinel nodes in carcinoma of the breast. *Journal of the American College of Surgeons*. Nov 2008;207(5):758-62. doi:10.1016/j.jamcollsurg.2008.06.341

53. Shimazu K, Tamaki Y, Taguchi T, Tsukamoto F, Kasugai T, Noguchi S. Intraoperative frozen section analysis of sentinel lymph node in breast cancer patients treated with neoadjuvant chemotherapy. *Annals of surgical oncology*. Jun 2008;15(6):1717-22. doi:10.1245/s10434-008-9831-3

54. Vanderveen KA, Ramsamooj R, Bold RJ. A prospective, blinded trial of touch prep analysis versus frozen section for intraoperative evaluation of sentinel lymph nodes in breast cancer. *Annals of surgical oncology*. Jul 2008;15(7):2006-11. doi:10.1245/s10434-008-9944-8

55. Horvath JW, Barnett GE, Jimenez RE, Young DC, Povoski SP. Comparison of intraoperative frozen section analysis for sentinel lymph node biopsy during breast cancer surgery for invasive lobular carcinoma and invasive ductal carcinoma. *World journal of surgical oncology*. Mar 24 2009;7:34. doi:10.1186/1477-7819-7-34

56. Krishnamurthy S, Meric-Bernstam F, Lucci A, et al. A prospective study comparing touch imprint cytology, frozen section analysis, and rapid cytokeratin immunostain for intraoperative evaluation of axillary sentinel lymph nodes in breast cancer. *Cancer*. Apr 1 2009;115(7):1555-62. doi:10.1002/cncr.24182

57. Langer I, Guller U, Berclaz G, et al. Accuracy of frozen section of sentinel lymph nodes: a prospective analysis of 659 breast cancer patients of the Swiss multicenter study. *Breast cancer research and treatment*. Jan 2009;113(1):129-36. doi:10.1007/s10549-008-9911-x

58. Tille JC, Egger JF, Devillaz MC, Vlastos G, Pelte MF. Frozen section in axillary sentinel lymph nodes for diagnosis of breast cancer micrometastasis. *Anticancer research*. Nov 2009;29(11):4711-6.

59. Upender S, Mohan H, Handa U, Attri AK. Intraoperative evaluation of sentinel lymph nodes in breast carcinoma by imprint cytology, frozen section and rapid immunohistochemistry. *Diagnostic cytopathology*. Dec 2009;37(12):871-5. doi:10.1002/dc.21120

60. van de Vrande S, Meijer J, Rijnders A, Klinkenbijl JH. The value of intraoperative frozen section examination of sentinel lymph nodes in breast cancer. *European journal of surgical oncology : the journal of the European Society of Surgical Oncology and the British Association of Surgical Oncology*. Mar 2009;35(3):276-80. doi:10.1016/j.ejso.2008.07.016

61. Cipolla C, Cabibi D, Fricano S, Vieni S, Gentile I, Latteri MA. The value of intraoperative frozen section examination of sentinel lymph nodes in surgical management of breast carcinoma. *Langenbeck's archives of surgery*. Aug 2010;395(6):685-91. doi:10.1007/s00423-009-0574-9

62. Geertsema D, Gobardhan PD, Madsen EV, et al. Discordance of intraoperative frozen section analysis with definitive histology of sentinel lymph nodes in breast cancer surgery: complementary axillary lymph node dissection is irrelevant for subsequent systemic therapy. *Annals of surgical oncology*. Oct 2010;17(10):2690-5. doi:10.1245/s10434-010-1052-x

63. Jaka RC, Zaveri SS, Somashekhar SP, Sureshchandra, Parameswaran RV. Value of frozen section and primary tumor factors in determining sentinel lymph node spread in early breast carcinoma. *Indian journal of surgical oncology*. Jan 2010;1(1):27-36. doi:10.1007/s13193-010-0008-8

64. Memar B, Sadeghi R, Ayati NK, et al. The value of touch imprint cytology and frozen section for intra- operative evaluation of axillary sentinel lymph nodes. *Polish journal of pathology : official journal of the Polish Society of Pathologists*. 2010;61(3):161-5.

65. Nofech-Mozes S, Hanna WM, Cil T, Quan ML, Holloway C, Khalifa MA. Intraoperative consultation for axillary sentinel lymph node biopsy: an 8-year audit. *International journal of surgical pathology*. Apr 2010;18(2):129-37. doi:10.1177/1066896909332114

66. Rubio IT, Aznar F, Lirola J, Peg V, Xercavins J. Intraoperative assessment of sentinel lymph nodes after neoadjuvant chemotherapy in patients with breast cancer. *Annals of surgical oncology*. Jan 2010;17(1):235-9. doi:10.1245/s10434-009-0695-y

67. Chan YH, Hung WK, Mak KL, Ying MW, Chan MC, Lui CY. Intra-operative assessment of axillary sentinel lymph nodes by frozen section-an observational study of 260 procedures. *Asian journal of surgery*. Apr 2011;34(2):81-5. doi:10.1016/s1015-9584(11)60024-9

68. Elezoğlu B, Tolunay Ş, Tasdelen I, GÖKgÖZ Ş. Histopathologic Characteristics of Sentinel Lymph Node Biopsy in Breast Carcinoma: Uludağ University Faculty of Medicine Experience. *Turkiye Klinikleri Journal of Medical Sciences*. 12/01 2011;31:1324-1329. doi:10.5336/medsci.2010-16770

69. Jamal MH, Rayment JH, Meguerditchian A, Doi SA, Meterissian S. Impact of the sentinel node frozen section result on the probability of additional nodal metastases as predicted by the MSKCC nomogram in breast cancer. *Japanese journal of clinical oncology*. Mar 2011;41(3):314-9. doi:10.1093/jjco/hyq225

70. Liu LC, Lang JE, Lu Y, et al. Intraoperative frozen section analysis of sentinel lymph nodes in breast cancer patients: a meta-analysis and single-institution experience. *Cancer*. Jan 15 2011;117(2):250-8. doi:10.1002/cncr.25606

71. Lumachi F, Norberto L, Zanella S, et al. Axillary node sampling in conjunction with sentinel node biopsy in patients with breast cancer. A prospective preliminary study. *Anticancer research*. Feb 2011;31(2):693-7.

72. Le Frere-Belda MA, Bats AS, Gillaizeau F, et al. Diagnostic performance of one-step nucleic acid amplification for intraoperative sentinel node metastasis detection in breast cancer patients. *International journal of cancer*. May 15 2012;130(10):2377-86. doi:10.1002/ijc.26291

73. Lumachi F, Marino F, Zanella S, Chiara GB, Basso SM. Touch imprint cytology and frozen-section analysis for intraoperative evaluation of sentinel nodes in early breast cancer. *Anticancer research*. Aug 2012;32(8):3523-6.

74. Safai A, Razeghi A, Monabati A, Azarpira N, Talei A. Comparing touch imprint cytology, frozen section analysis, and cytokeratin immunostaining for intraoperative evaluation of axillary sentinel lymph nodes in breast cancer. *Indian journal of pathology & microbiology*. Apr-Jun 2012;55(2):183-6. doi:10.4103/0377-4929.97859

75. Stovgaard ES, Tvedskov TF, Laenkholm AV, Balslev E. Cytokeratin on frozen sections of sentinel node may spare breast cancer patients secondary axillary surgery. *Pathology research international*. 2012;2012:802184. doi:10.1155/2012/802184

76. Taffurelli M, Montroni I, Santini D, et al. Effectiveness of sentinel lymph node intraoperative examination in 753 women with breast cancer: are we overtreating patients? *Annals of surgery*. May 2012;255(5):976-80. doi:10.1097/SLA.0b013e31824def4e

77. Wang YS, Ou-yang T, Wu J, et al. Comparative study of one-step nucleic acid amplification assay, frozen section, and touch imprint cytology for intraoperative assessment of breast sentinel lymph node in Chinese patients. *Cancer science*. Nov 2012;103(11):1989-93. doi:10.1111/cas.12001

78. Arlicot C, Louarn AL, Arbion F, et al. Evaluation of the two intraoperative examination methods for sentinel lymph node assessment: a multicentric and retrospective study on more than 2,000 nodes. *Anticancer research*. Mar 2013;33(3):1045-52.

79. Ballehaninna UK, Chamberlain RS. Utility of intraoperative frozen section examination of sentinel lymph nodes in ductal carcinoma in situ of the breast. *Clinical breast cancer*. Oct 2013;13(5):350-8. doi:10.1016/j.clbc.2013.02.013

80. Han A, Moon HG, Kim J, et al. Reliability of sentinel lymph node biopsy after neoadjuvant chemotherapy in breast cancer patients. *Journal of breast cancer*. Dec 2013;16(4):378-85. doi:10.4048/jbc.2013.16.4.378

81. Hashmi AA, Faridi N, Khurshid A, et al. Accuracy of frozen section analysis of sentinel lymph nodes for the detection of Asian breast cancer micrometastasis - experience from Pakistan. *Asian Pacific journal of cancer prevention : APJCP*. 2013;14(4):2657-62. doi:10.7314/apjcp.2013.14.4.2657

82. Lim J, Govindarajulu S, Sahu A, Ibrahim N, Magdub S, Cawthorn S. Multiple Step-section Frozen Section sentinel lymph node biopsy--a review of 717 patients. *Breast (Edinburgh, Scotland)*. Oct 2013;22(5):639-42. doi:10.1016/j.breast.2013.07.044

83. Lu Q, Tan EY, Ho B, et al. Achieving breast cancer surgery in a single setting with intraoperative frozen section analysis of the sentinel lymph node. *Clinical breast cancer*. Apr 2013;13(2):140-5. doi:10.1016/j.clbc.2012.11.005

84. Moatasim A, Mujtaba S, Faridi N. Intraoperative frozen section analysis of sentinel lymph nodes in breast carcinoma patients in a tertiary hospital in Pakistan. *International journal of surgery (London, England)*. 2013;11(3):253-8. doi:10.1016/j.ijsu.2013.01.007

85. Barakat FH, Sulaiman I, Sughayer MA. Reliability of frozen section in breast sentinel lymph node examination. *Breast cancer (Tokyo, Japan)*. Sep 2014;21(5):576-82. doi:10.1007/s12282-012-0431-5

86. Jara-Lazaro AR, Hussain IH, Thike AA, et al. Assessment of suitability of the one step nucleic acid amplification (OSNA) assay as an intraoperative procedure for detection of metastasis in sentinel lymph nodes of breast cancer. *Journal of clinical pathology*. Dec 2014;67(12):1032-7. doi:10.1136/jclinpath-2014-202361

87. Poling JS, Tsangaris TN, Argani P, Cimino-Mathews A. Frozen section evaluation of breast carcinoma sentinel lymph nodes: a retrospective review of 1,940 cases. *Breast cancer research and treatment*. Nov 2014;148(2):355-61. doi:10.1007/s10549-014-3161-x

88. Wang YS, Liu YH, Tao OY, et al. GeneSearch BLN Assay could replace frozen section and touch imprint cytology for intra-operative assessment of breast sentinel lymph nodes. *Breast cancer (Tokyo, Japan)*. Sep 2014;21(5):583-9. doi:10.1007/s12282-012-0437-z

89. Nowikiewicz T, Srutek E, Zegarski W. Application of immunohistochemistry for detection of metastases in sentinel lymph nodes of non-advanced breast cancer patients. *Polish journal of pathology : official journal of the Polish Society of Pathologists*. Mar 2015;66(1):22-9. doi:10.5114/pjp.2015.51149

90. Somashekhar SP, Naikoo ZA, Zaveri SS, et al. Intraoperative Frozen Section Evaluation of Sentinel Lymph Nodes in Breast Carcinoma: Single-Institution Indian Experience. *The Indian journal of surgery*. Dec 2015;77(Suppl 2):335-40. doi:10.1007/s12262-013-0827-2

91. Vohra LM, Gulzar R, Saleem O. INTRA OPERATIVE FROZEN EXAMINATION OF SENTINEL LYMPH NODE IN BREAST CANCER. *Journal of Ayub Medical College, Abbottabad : JAMC*. Jan-Mar 2015;27(1):40-4.

92. Wong J, Yong WS, Thike AA, et al. False negative rate for intraoperative sentinel lymph node frozen section in patients with breast cancer: a retrospective analysis of patients in a single Asian institution. *Journal of clinical pathology*. Jul 2015;68(7):536-40. doi:10.1136/jclinpath-2014-202799

93. Abuoglu HH, Gunay E, Sunamak O, Yigitbasi MR. Diagnostic Value of Frozen Section in Patients with Non-Palpable Breast Lesions. *Chirurgia (Bucharest, Romania : 1990)*. Nov-Dec 2016;111(6):500-504. doi:10.21614/chirurgia.111.6.500

94. Qiao G, Cong Y, Zou H, et al. False-negative Frozen Section of Sentinel Lymph Node Biopsy in a Chinese Population with Breast Cancer. *Anticancer research*. Mar 2016;36(3):1331-7.

95. Tan J, Joblin L, Davenport E. Accuracy of frozen sections for breast cancer sentinel lymph node biopsies within a peripheral New Zealand hospital. *The New Zealand medical journal*. Mar 11 2016;129(1431):46-50.

96. Ahadi M, Zham H, Kiafar M, et al. Compatibility of Intraoperative Frozen Section Analysis with Permanent Section Analysis of Sentinel Lymph Nodes in Breast Cancer. Research Article. *Int J Cancer Manag*. 2017;10(9):e11571. doi:10.5812/ijcm.11571

97. Ballal DS, Rakshit SH, Somashekhar SP, Arunkumar N. Evaluation of Intraoperative Frozen Section with Final Histopathology Results for Sentinel Lymph Node Biopsy in Breast Cancer. *Indian Journal of Gynecologic Oncology*. 2017/09/21 2017;15(4):54. doi:10.1007/s40944-017-0145-x

98. Espinosa-Bravo M, Navarro-Cecilia J, Ramos Boyero M, et al. Intraoperative assessment of sentinel lymph node by one-step nucleic acid amplification in breast cancer patients after neoadjuvant treatment reduces the need for a second surgery for axillary lymph node dissection. *Breast (Edinburgh, Scotland)*. Feb 2017;31:40-45. doi:10.1016/j.breast.2016.10.002

99. Petropoulou T, Kapoula A, Mastoraki A, et al. Imprint cytology versus frozen section analysis for intraoperative assessment of sentinel lymph node in breast cancer. *Breast cancer (Dove Medical Press)*. 2017;9:325-330. doi:10.2147/bctt.s130987

100. Russo L, Betancourt L, Romero G, et al. Frozen section evaluation of sentinel lymph nodes in breast carcinoma: a retrospective analysis. *Ecancermedicalscience*. 2017;11:774. doi:10.3332/ecancer.2017.774

101. Sun L, Chen G, Zhou Y, et al. Clinical significance of MSKCC nomogram on guiding the application of touch imprint cytology and frozen section in intraoperative assessment of breast sentinel lymph nodes. *Oncotarget*. Sep 29 2017;8(44):78105-78112. doi:10.18632/oncotarget.17490

102. Lai SK, Masir N, Md Pauzi SH. Intraoperative frozen section sentinel lymph node assessment in breast cancer: A tertiary institution experience. *The Malaysian journal of pathology*. Aug 2018;40(2):121-128.

103. Lombardi A, Nigri G, Maggi S, et al. Role of frozen section in sentinel lymph node biopsy for breast cancer in the era of the ACOSOG Z0011 and IBCSG 23-10 trials. *The surgeon : journal of the Royal Colleges of Surgeons of Edinburgh and Ireland*. Aug 2018;16(4):232-236. doi:10.1016/j.surge.2017.11.003

104. Grabenstetter A, Moo TA, Hajiyeva S, et al. Accuracy of Intraoperative Frozen Section of Sentinel Lymph Nodes After Neoadjuvant Chemotherapy for Breast Carcinoma. *The American journal of surgical pathology*. Oct 2019;43(10):1377-1383. doi:10.1097/pas.0000000000001311

105. Houpu Y, Fei X, Yang Y, et al. Use of Memorial Sloan Kettering Cancer Center nomogram to guide intraoperative sentinel lymph node frozen sections in patients with early breast cancer. *Journal of surgical oncology*. Sep 2019;120(4):587-592. doi:10.1002/jso.25638

106. Wong W, Rubenchik I, Nofech-Mozes S, et al. Intraoperative Assessment of Sentinel Lymph Nodes in Breast Cancer Patients Post-Neoadjuvant Therapy. *Technology in cancer research & treatment*. Jan 1 2019;18:1533033818821104. doi:10.1177/1533033818821104

107. Yoon KH, Park S, Kim JY, et al. Is the frozen section examination for sentinel lymph node necessary in early breast cancer patients? *Annals of surgical treatment and research*. Aug 2019;97(2):49-57. doi:10.4174/astr.2019.97.2.49

108. Cipolla C, Graceffa G, Cabibi D, et al. Current Role of Intraoperative Frozen Section Examination of Sentinel Lymph Node in Early Breast Cancer. *Anticancer research*. Mar 2020;40(3):1711-1717. doi:10.21873/anticanres.14124

109. Shojaee L, Abedinnegad S, Nafisi N, et al. Sentinel Node Biopsy in Early Breast Cancer Patients with Palpable Axillary Node. *Asian Pacific journal of cancer prevention : APJCP*. Jun 1 2020;21(6):1631-1636. doi:10.31557/apjcp.2020.21.6.1631

110. Cotarelo CL, Zschock-Manus A, Schmidt M, et al. Improved detection of sentinel lymph node metastases allows reliable intraoperative identification of patients with extended axillary lymph node involvement in early breast cancer. *Clinical & experimental metastasis*. Feb 2021;38(1):61-72. doi:10.1007/s10585-020-10065-9
